# Supplementary material for: Advancing intercalation strategies in layered hybrid perovskites by bringing together synthesis and simulations
Source: Nat Commun. 2025 Jul 1;16:5549. doi: 10.1038/s41467-025-60880-5 (PMC12215875; doi:10.1038/s41467-025-60880-5)
Supplement: Supplementary file 1 — Supplementary Information [file 41467_2025_60880_MOESM1_ESM.pdf]

Supplementary Information

Advancing Intercalation Strategies in Layered Hybrid Perovskites by Bringing Together Synthesis and Simulations

Lin-Jie Yang, Wenye Xuan, Sara Henda, Shaoyang Wang, Sai Kiran Rajendran, David B. Cordes, David N. Miller, Alexandra M. Z. Slawin, Lethy Krishnan Jagadamma, Hamid Ohadi, Hsin-Yi Tiffany Chen, Matthew S. Dyer and Julia L. Payne

Supplementary Table, Figures and Discussion: Results from DFT Calculations

Supplementary Table 1: Structural parameters obtained from calculations for intercalated perovskites, $[H_3N(CH_2)_mNH_3]PbX_4{\cdot}X_2$ where m= 5 -10 and X = Cl, Br, I.

m number	Halide	Guest Molecules	Θ_1 (°)	Θ_2 (°)	$\lvert \Delta D \rvert$ (Å)
5	Cl	Cl_2	170.5874	170.0335	0.1447
5	Br	Cl_2	173.6734	172.4234	0.12402
5	I	Cl_2	167.3071	175.585	1.31981
5	Cl	Br_2	168.2712	177.1391	0.04997
5	Br	Br_2	172.0106	175.8371	0.06278
5	I	Br_2	173.2671	173.897	0.59077
5	Cl	I_2	155.9635	174.2919	0.22618
5	Br	I_2	168.5068	170.8227	0.15944
5	I	I_2	171.3958	169.3539	0.2763
6	Cl	Cl_2	175.7588	179.7488	0.22608
6	Br	Cl_2	177.0997	178.6205	0.08025
6	I	Cl_2	177.8401	178.5453	0.018
6	Cl	Br_2	176.2829	178.0154	0.03649
6	Br	Br_2	176.7938	176.7181	0.04077
6	I	Br_2	177.9719	176.6535	0.02325
6	Cl	I_2	174.1605	173.5493	0.12964
6	Br	I_2	174.9178	171.8316	0.08956
6	I	I_2	174.6887	165.9887	0.1051
7	Cl	Cl_2	173.9305	177.2409	1.52696
7	Br	Cl_2	178.9363	177.9988	0.67451
7	I	Cl_2	177.8183	177.2597	0.54094
7	Cl	Br_2	171.3596	175.3095	1.10331
7	Br	Br_2	178.2143	176.6882	0.25595
7	I	Br_2	177.9302	178.7012	0.10376
7	Cl	I_2	170.8485	166.2028	0.14598
7	Br	I_2	171.2043	166.0728	0.16084
7	I	I_2	171.7882	168.0075	0.09469
8	Cl	Cl_2	177.2071	176.8298	1.01374
8	Br	Cl_2	178.3378	172.8224	0.9383
8	I	Cl_2	176.6536	178.6361	0.38197
8	Cl	Br_2	178.3201	173.5112	0.50079
8	Br	Br_2	176.6407	174.3654	0.15743
8	I	Br_2	177.1367	177.6077	0.09985
8	Cl	I_2	176.6224	173.954	0.00316
8	Br	I_2	175.3091	173.1223	0.0021
8	I	I_2	174.7772	173.795	0.06533
9	Cl	Cl_2	175.3431	160.2743	2.05691
9	Br	Cl_2	176.84	156.4957	1.47204
9	I	Cl_2	176.0858	153.6217	1.29585
9	Cl	Br_2	170.294	163.0317	1.1573
9	Br	Br_2	174.185	158.3164	0.96291
9	I	Br_2	172.7755	159.8663	0.59421
9	Cl	I_2	167.1303	167.1477	0.01471

9	**Br**	**I_2**	167.0496	166.9526	0.00116
9	**I**	**I_2**	166.1702	166.1805	6.20E-04
10	**Cl**	**Cl_2**	178.7719	148.0818	5.17466
10	**Br**	**Cl_2**	179.243	160.9135	4.36986
10	**I**	**Cl_2**	179.4351	172.6982	3.4636
10	**Cl**	**Br_2**	178.1164	160.915	4.2071
10	**Br**	**Br_2**	178.2119	163.9078	3.73111
10	**I**	**Br_2**	179.3005	173.0294	2.99167
10	**Cl**	**I_2**	175.4778	163.2836	3.27463
10	**Br**	**I_2**	176.936	163.384	3.06424
10	**I**	**I_2**	178.7449	164.7404	2.62038

The values from Supplementary Table 1 are summarised in Supplementary Figures 1 – 3. Compositions that could be obtained in single crystal form with publication quality CIFs are highlighted in the table in blue. Although calculations show that $[H_3N(CH_2)_8NH_3]PbBr_4{\cdot}Br_2$ exhibits physically sensible structural parameters, we were unable to prepare good quality single crystals of this composition and it is highlighted in red. $[H_3N(CH_2)_6NH_3]PbI_4{\cdot}Br_2$ and $[H_3N(CH_2)_7NH_3]PbI_4{\cdot}Br_2$ are highlighted in yellow, as they show the lowest bandgap amongst all perovskites investigated in this study. However, these samples cannot be obtained as a bulk crystalline form due to the halide exchange between the intercalated halogen molecules and halide ions in the inorganic layers.

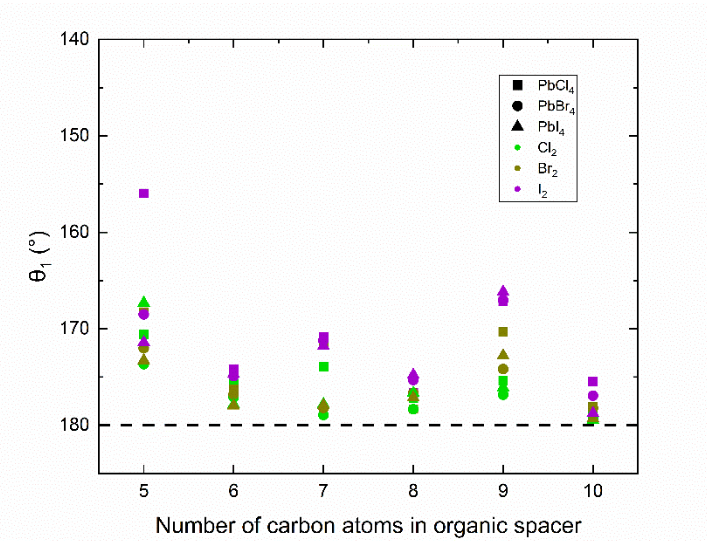

Supplementary Figure 1: Calculated bond angle (Θ_1) between covalent bond of X_2 and one proximal (halogen-bonded) X-site anion from inorganic layers for $[H_3N(CH_2)_mNH_3]PbX_4{\cdot}X_2$ perovskites (where m = 5-10 and X = Cl, Br or I).

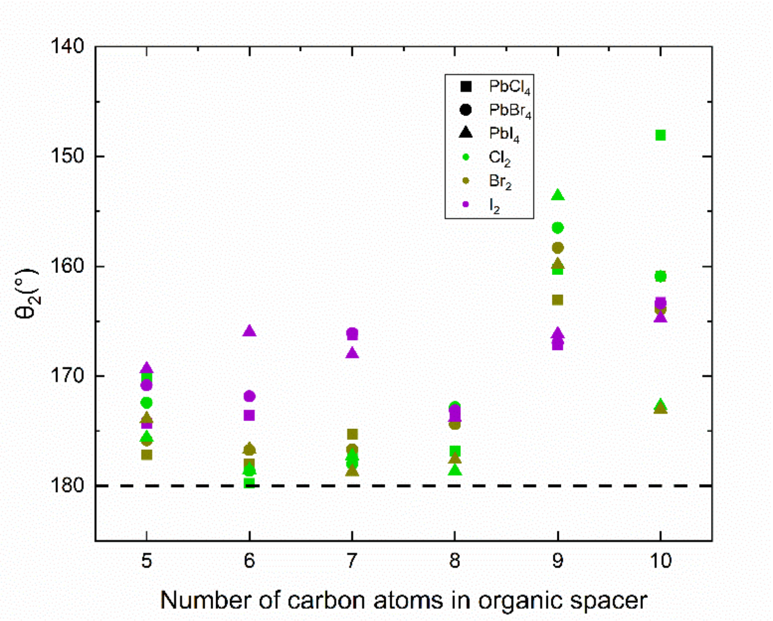

*Supplementary Figure 2: Calculated bond angle (**Θ_2**) between the covalent bond of X_2 and the other proximal (halogen bonded) vertical X-site anion from inorganic layers, for $[H_3N(CH_2)_mNH_3]PbX_4{\cdot}X_2$ perovskites (where m = 5-10 and X = Cl, Br or I).*

In Supplementary Figures 1 and 2, different colours are used for different intercalated molecules. For example, according to Supplementary Figure 2, the **Θ_2** of hypothetical sample $[H_3N(CH_2)_6NH_3]PbCl_4{\cdot}Cl_2$ is 180°.

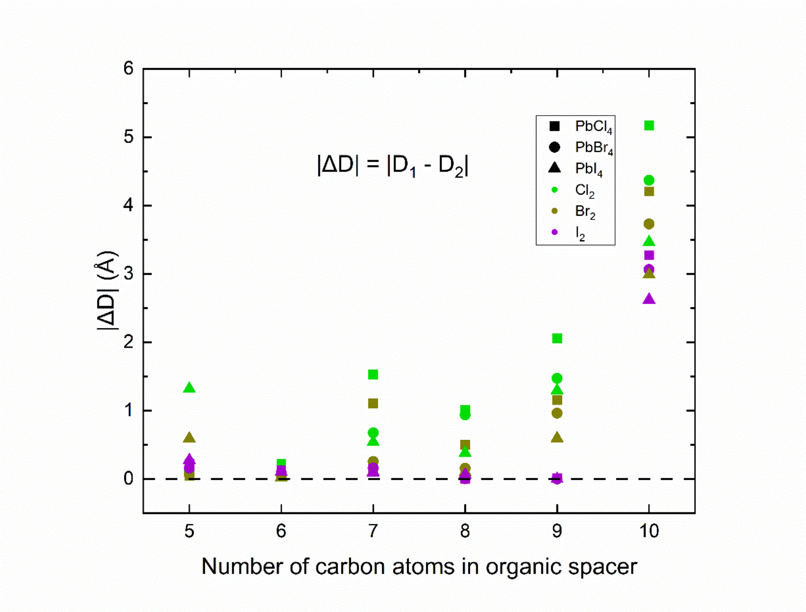

Supplementary Figure 3: Calculated bond length differences, $|\triangle D|$ (where $|\Delta \mathbf{D}|=|\mathbf{D_1}$-$\mathbf{D_2}|$ between the two halogen bonds stemming from the same X_2 molecules, for $[H_3N(CH_2)_mNH_3]PbX_4{\cdot}X_2$ perovskites (where m = 5-10 and X = Cl, Br or I).

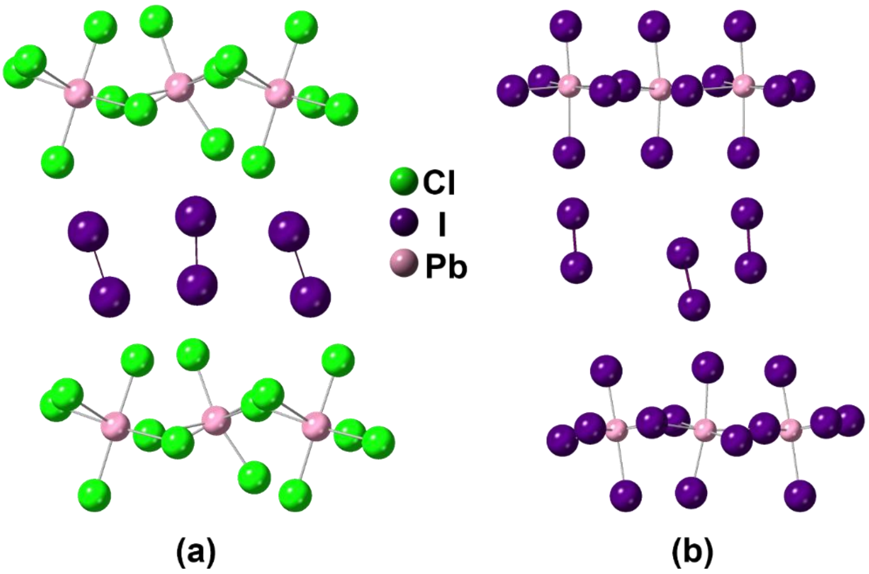

Supplementary Figure 4: Simulated crystal structure from DFT for (a) $[H_3N(CH_2)_5NH_3]PbCl_4{\cdot}I_2$ and (b) $[H_3N(CH_2)_{10}NH_3]PbI_4{\cdot}I_2$. Lead (pink), chlorine (green) and iodine (purple) and the $[H_3N(CH_2)_mNH_3]^{2+}$ cations are omitted for clarity.

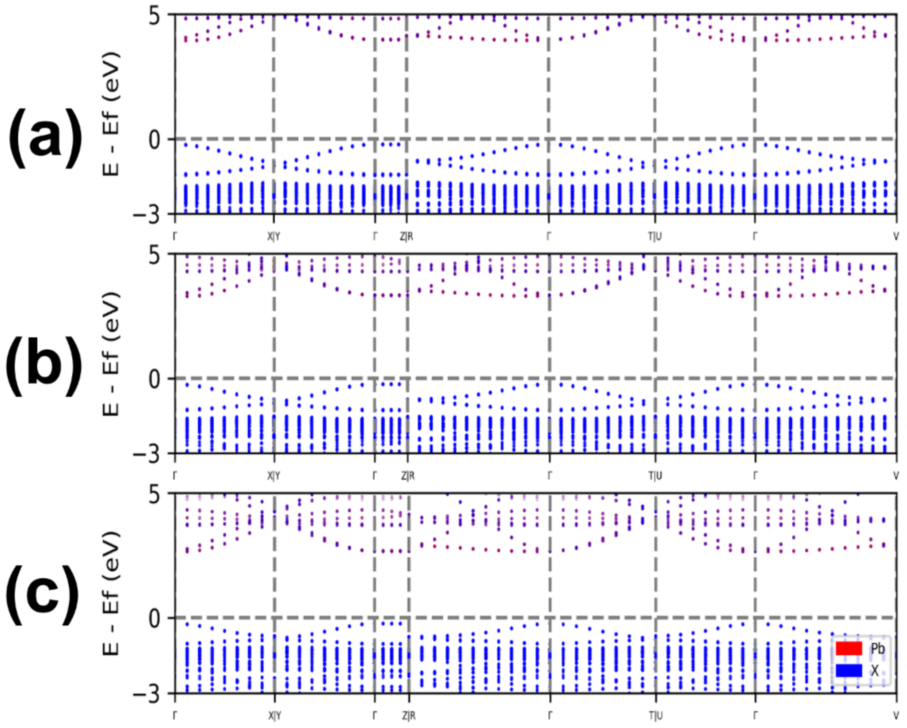

Supplementary Figure 5: Band structure of parent ($[H_3N(CH_2)_mNH_3]PbX_4$) layered hybrid perovskites (a) $[H_3N(CH_2)_6NH_3]PbCl_4$, (b) $[H_3N(CH_2)_6NH_3]PbBr_4$, and (c) $[H_3N(CH_2)_6NH_3]PbI_4$, calculated using the HSE06 functional.

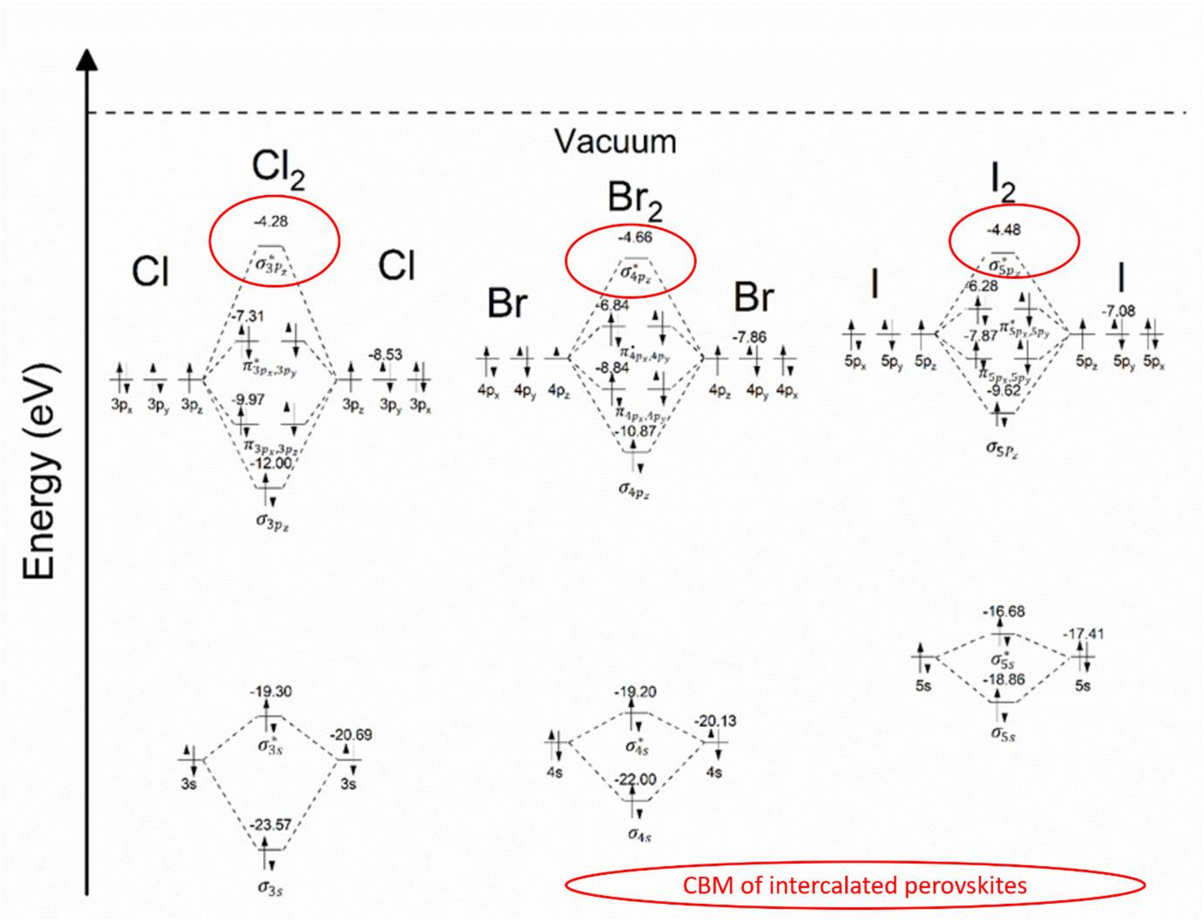

Supplementary Figure 6: Calculated molecular orbital energy level diagram for Cl_2, Br_2 and I_2

As shown in the main text (Figure 2c), we can see that the computational structure parameters are in excellent agreement with those obtained from single crystal XRD data, especially for the structures with centrosymmetric space groups such as $[H_3N(CH_2)_6NH_3]PbBr_4{\cdot}Br_2$ and $[H_3N(CH_2)_9NH_3]PbI_4{\cdot}I_2$. Here, there is a negligible $|\triangle \boldsymbol{D}|$ and $\boldsymbol{\Theta_1}$ and $\boldsymbol{\Theta_2}$ are almost the same. From the -structural data, we noticed that $|\triangle \boldsymbol{D}|$ has a maximum < 0.1 Å and $\boldsymbol{\Theta}$ has a minimum $> 171°$. Such values can be used as a basis for the further development and optimisation of intercalated materials in the future.

Supplementary Methods

Supplementary Table 2: Reagents and reaction conditions for the syntheses of $[H_3N(CH_2)_mNH_3]PbX_4$ and $[H_3N(CH_2)_mNH_3]PbX_4{\cdot}X_2$ (m =7-9, X = Br, I).

Single Crystal Growth			
**Intercalated Samples**	**Reagents**	**Autoclave Reaction Temperature and Duration**	**Crystal Description**
**$[H_3N(CH_2)_7NH_3]PbBr_4{\cdot}Br_2$**	$PbBr_2$ (0.734 g, 2 mmol)<br>$H_2N(CH_2)_7NH_2$ (0.264 g, 2 mmol)<br>HBr (7 mL)<br>Br_2 (5 mL)	120 °C (8 hours)<br>+<br>80 °C (24 hours)	orange prism
**$[H_3N(CH_2)_7NH_3]PbBr_4{\cdot}IBr$**	$PbBr_2$ (0.734 g, 2 mmol)<br>$H_2N(CH_2)_7NH_2$ (0.264 g, 2 mmol)<br>HBr (10 mL)<br>IBr (0.45 g, 2.17 mmol)	120 °C (8 hours)<br>+<br>80 °C (24 hours)	yellow prism
**$[H_3N(CH_2)_7NH_3]PbI_4{\cdot}I_2$**	PbI_2 (0.922 g, 2 mmol)<br>$H_2N(CH_2)_7NH_2$ (0.264 g, 2 mmol)<br>HI (12 mL)<br>I_2 (0.55 g, 2.16 mmol)	160 °C (8 hours)<br>+<br>80 °C (24 hours)	red prism
**$[H_3N(CH_2)_8NH_3]PbBr_4{\cdot}I_2$**	$PbBr_2$ (0.734 g, 2 mmol)<br>$H_2N(CH_2)_8NH_2$ (0.292 g, 2 mmol)<br>HBr (10 mL)<br>I_2 (0.55 g, 2.16 mmol)	140 °C (8 hours)<br>+<br>80 °C (24 hours)	yellow platelet
**$[H_3N(CH_2)_8NH_3]PbI_4{\cdot}I_2$**	PbI_2 (0.922 g, 2 mmol)<br>$H_2N(CH_2)_8NH_2$ (0.292 g, 2 mmol)<br>HI (12 mL)<br>I_2 (0.55 g, 2.16 mmol)	160 °C (8 hours)<br>+<br>80 °C (24 hours)	red platelet
**$[H_3N(CH_2)_9NH_3]PbI_4{\cdot}I_2$**	PbI_2 (0.922 g, 2 mmol)<br>$H_2N(CH_2)_9NH_2$ (0.321 g, 2 mmol)<br>HI (12 mL)<br>I_2 (0.55 g, 2.16 mmol)	160 °C (8 hours)<br>+<br>80 °C (24 hours)	red prism

Supplementary Tables, Discussion and Figures: Crystallography

Supplementary Table 3: Crystallographic and refinement details for 6 new intercalated perovskites $[H_3N(CH_2)_mNH_3]PbX_4{\cdot}X_2$ and $[H_3N(CH_2)_mNH_3]PbX_4{\cdot}XX$ where m = 7 to 9 and X = Br or I.

Sample	$[H_3N(CH_2)_7NH_3]PbBr_4{\cdot}Br_2$	$[H_3N(CH_2)_7NH_3]PbBr_4{\cdot}IBr$	$[H_3N(CH_2)_7NH_3]PbI_4{\cdot}I_2$	$[H_3N(CH_2)_8NH_3]PbBr_4{\cdot}I_2$	$[H_3N(CH_2)_8NH_3]PbI_4{\cdot}I_2$	$[H_3N(CH_2)_9NH_3]PbI_4{\cdot}I_2$
CCDC Code	2340844	2340845	2340846	2340847	2340848	2340849
Formula	$PbBr_6C_7N_2H_{20}$	$PbBr_5I_1C_7N_2H_{20}$	$PbI_6C_7N_2H_{20}$	$PbBr_4I_2C_8N_2H_{22}$	$PbI_6C_8N_2H_{22}$	$PbI_6C_9N_2H_{24}$
Formula Weight	818.90	865.89	1100.84	926.90	1114.86	1128.89
Crystal Description	Orange Prism	Yellow Prism	Red Prism	Yellow Platelet	Red Platelet	Red Prism
Crystal Size (mm^3)	0.15 × 0.11 × 0.04	0.26 ×0.16 × 0.08	0.09 ×0.08 × 0.07	0.03 × 0.03 × 0.01	0.08 × 0.05 × 0.01	0.18 × 0.03 × 0.02
Temperature (K)	173	298	298	173	173	298
Crystal System	Orthorhombic	Orthorhombic	Orthorhombic	Monoclinic	Monoclinic	Monoclinic
Space group	$P2_12_12_1$	*Pnma*	$P2_12_12_1$	$P2_1/c$	$P2_1/n$	$C2/c$
a (Å)	7.9443(3)	8.0388(5)	8.5048(5)	8.4006(9)	8.7303(6)	29.8750(2)
b (Å)	8.3591(3)	29.634(2)	8.5632(6)	8.1555(8)	8.6637(6)	9.0315(6)
c (Å)	28.6132(10)	8.1560(5)	31.1090(2)	30.006(3)	29.609(2)	8.8086(6)
β (°)				93.078(3)	90.959(2)	90.8490(10)
V ($Å^3$)	1900.12(12)	1942.9(2)	2265.6(2)	2052.8(4)	2239.2(3)	2376.4(2)
Z	4	4	4	4	4	4
ρ_{calc} (g/cm^3)	2.863	2.960	3.227	2.999	3.307	3.155
μ (mm^{-1})	21.479	20.538	15.609	19.000	15.796	14.886
F(000)	1472	1544	1904	1648	1936	1968
Reflections collected	25175	18352	23263	23968	22671	12425
Independent reflections (R_{int})	4486 (0.0549)	2270 (0.1304)	5185 (0.0790)	3756 (0.0528)	5130 (0.2194)	2740 (0.1098)
Parameters, Restraints	148, 126	77, 64	148, 134	156, 270	157, 183	85, 95

Goodness-of-fit on F^2	1.048	1.202	1.091	1.046	1.016	1.031
R_1	0.0478	0.1603	0.0947	0.0379	0.2067	0.1536
R_1 [I>2sigma (I)]	0.0370	0.1246	0.0485	0.0302	0.0844	0.0743
wR_2	0.1001	0.3464	0.1124	0.0762	0.2337	0.2322
wR_2 [I>Sigma(I)]	0.0964	0.3325	0.0985	0.0785	0.1839	0.1903
Largest diff. peak and hole (e/Å^3)	1.882 and -1.705	3.218 and -2.226	1.002 and -1.566	1.149 and -1.742	2.751 and -2.766	2.218 and -2.331

We were able to synthesise single crystals of $[H_3N(CH_2)_8NH_3]PbBr_4{\cdot}Br_2$. However, crystal quality was poor and while the lead-halide framework appeared to be ordered, both the Br_2 molecules and the $[H_3N(CH_2)_8NH_3]^{2+}$cations showed poor ordering. From what can be seen in the structure, the halogen-bonding Br···Br distances (***D₁*** and ***D₂***) are large relative to other intercalated compounds, suggesting that the halogen bond is long and therefore weak. This enables the Br_2 molecules to vibrate within the structure, in turn forcing the $[H_3N(CH_2)_8NH_3]^{2+}$cation to alter its conformation to accommodate this, resulting in the observed minimal order. We believe this indicates that *m* = 8 is the 'upper limit' for stable intercalation of Br_2 molecules between lead bromide frameworks.

Supplementary Table 4: Key structural parameters of seven intercalated perovskites $[H_3N(CH_2)_mNH_3]PbX_4{\cdot}X_2$ and $[H_3N(CH_2)_mNH_3]PbX_4{\cdot}XX$, obtained from single crystal X-ray diffraction.

Key structural parameters from single crystal XRD									
m	***X* (halide anion)**	**X_2 (halogen molecule)**	**D_h (Å)**	**D_1 (Å)**	**D_2 (Å)**	**$D_{X\text{-}X}$ (Å)**	**Θ_1 (°)**	**Θ_2 (°)**	**D_L (Å)**
6	Br	Br_2	8.466	3.066		2.334	177.531		14.101
7	Br	Br_2	8.634	3.197	3.114	2.334	174.489	179.034	14.307
7	Br	IBr	8.982	3.169		2.667	175.196		14.817
7	I	I_2	9.458	3.397	3.343	2.746	174.472	175.126	15.555
8	Br	I_2	9.195	3.253	3.257	2.710	173.127	176.178	14.978
8	I	I_2	9.478	3.404	3.370	2.742	171.470	176.157	14.802
9	I	I_2	9.708	3.523		2.726	172.025		14.936

There is a slight displacement of lead atoms in the $[PbBr_4]_\infty$ layers in $[H_3N(CH_2)_7NH_3]PbBr_4{\cdot}Br_2$. $[H_3N(CH_2)_8NH_3]PbI_4{\cdot}I_2$ is best described in space group $P2_1/n$, which others have shown to be more unusual for layered hybrid perovskites.[1] In $[H_3N(CH_2)_8NH_3]PbBr_4{\cdot}I_2$, the $[H_3N(CH_2)_8NH_3]^{2+}$cation adopts a more twisted conformation, due to the short $\boldsymbol{D_h}$, $\boldsymbol{D_1}$ and $\boldsymbol{D_2}$. This results in one C-N bond being rotated symmetrically at both of ends of the carbon backbone. $[H_3N(CH_2)_9NH_3]PbI_4{\cdot}I_2$ shows a mirror plane through the central carbon atom in the chain (C5).

Powder X-ray Diffraction of Polycrystalline Samples

Powder X-ray diffraction was used to check phase purity of all samples prepared. Fitting the PXRD data using Le Bail, Pawley or Rietveld refinement is a much better way of testing whether structural models obtained from single crystal X-ray diffraction data are in agreement with PXRD data. We note that only phase identification quality PXRD data were collected, rather than Rietveld quality data. As a result, in most cases, we were unable to reliably refine realistic atomic displacement parameters when performing Rietveld refinement. For some samples, we also encountered some problems in fitting the data due to preferred orientation or poor powder averaging statistics. The samples were prepared in highly crystalline forms, but due to the organic-inorganic metal halides being much softer than other inorganic materials typically characterised by PXRD, such as inorganic oxides, grinding of samples before PXRD data collection can result in a degradation of crystallinity or deintercalation. As a result, sometimes we have PXRD data which show preferred orientation or poor powder averaging statistics, which can make it difficult to fit the data. Another factor which can cause difficulties in fitting the PXRD data (collected at room temperature) is the possibility of phase transitions in the materials between 173 K (temperature used for single crystal data collection) and 298 K (temperature used for PXRD data collection). We also note that in some samples an iron peak was observed in the PXRD pattern due to the sample holder. Fits to the PXRD data are given below, along with unit cell parameters and a list of the parameters that were refined. As can be seen in Figures S8-13, there is reasonable agreement between the experimental PXRD pattern collected at room temperature and the calculated PXRD pattern following Pawley or Rietveld refinements. $[H_3N(CH_2)_7NH_3]PbBr_4{\cdot}IBr$ showed a small proportion of another phase in its PXRD pattern and this is modelled in the Pawley refinement as a small proportion of the parent phase, $[H_3N(CH_2)_7NH_3]PbBr_4$ (Supplementary Figure 8).[2,3] $[H_3N(CH_2)_8NH_3]PbBr_4{\cdot}I_2$ also showed peaks

associated with another phase, however these did not match the parent phase. Instead, they showed good agreement with a monoclinic *C*-centred phase that had been apparent during attempted SCXRD data collections on this compound at room temperature (Supplementary Figure 10).

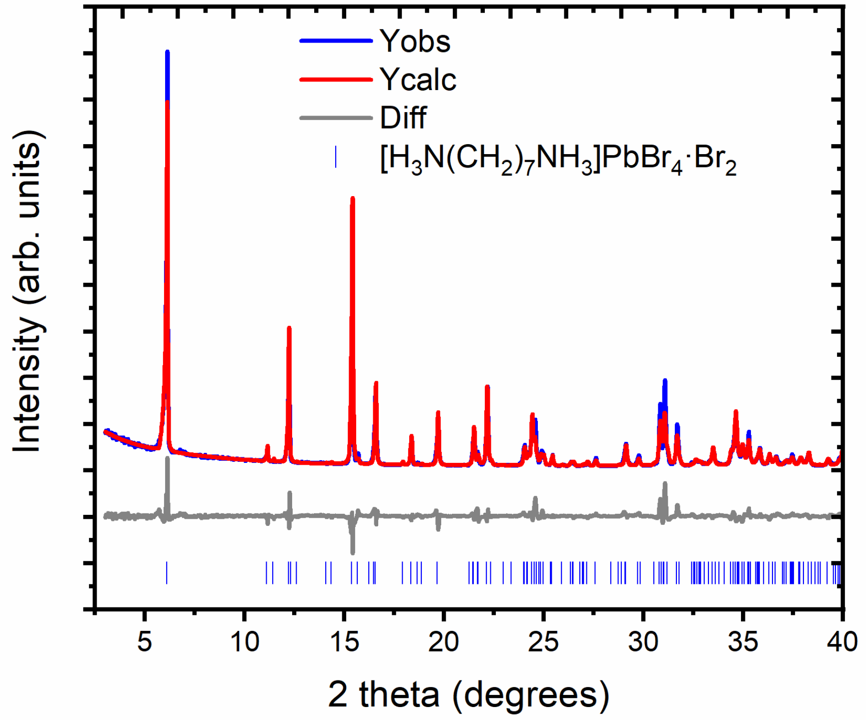

Supplementary Figure 7: Rietveld fit for [$H_3N(CH_2)_7NH_3$]$PbBr_4{\cdot}Br_2$. a = 8.0259(3) Å, b = 8.2729(4) Å, c = 29.0141(13) Å and volume = 1926.49(15) Å^3. R_{wp} = 11.795%. 12 background parameters, a term for peak asymmetry, profile parameters, sample displacement, unit cell parameters, atomic coordinates and individual U_{iso} for Pb and Br were refined.

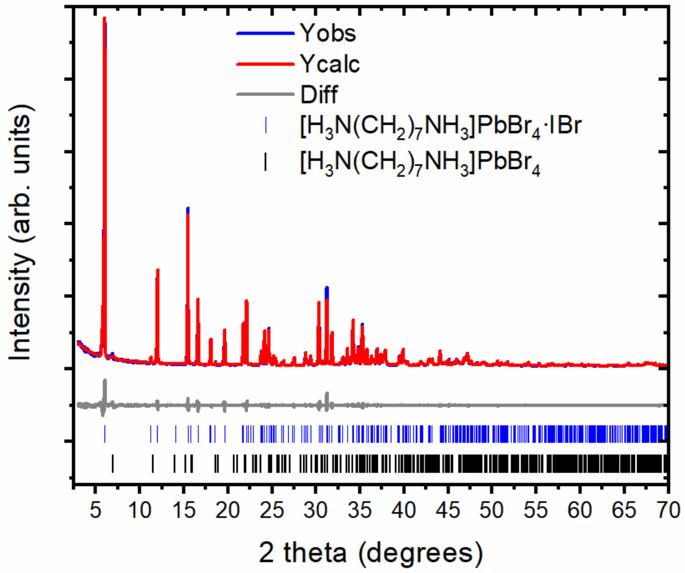

Supplementary Figure 8: Pawley fit for $[H_3N(CH_2)_7NH_3]PbBr_4·IBr$ with a small amount of the parent $[H_3N(CH_2)_7NH_3]PbBr_4$ impurity phase. The unit cell parameters of $[H_3N(CH_2)_7NH_3]PbBr_4·IBr$ are a = 8.03974(18) Å, b = 29.4684(6) Å, c = 8.1699(2) Å and volume = 1935.61(8) Å^3. The unit cell parameters of $[H_3N(CH_2)_7NH_3]PbBr_4$ were a = 25.29(5) Å, b = 8.061(18) Å, c = 8.09(3) Å and β = 91.52(17) °. R_{wp} = 6.807%. Pawley refinement, where 12 background parameters, asymmetry, profile parameters, sample displacement and unit cell parameters were refined.

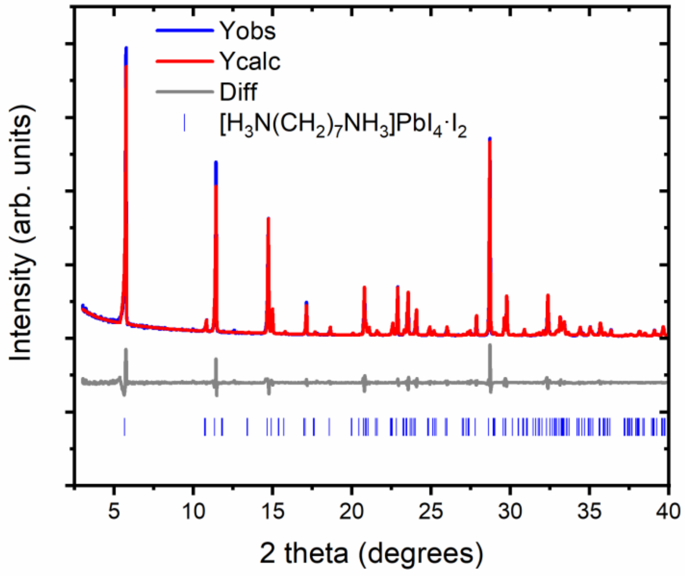

Supplementary Figure 9: Pawley fit for $[H_3N(CH_2)_7NH_3]PbI_4·I_2$. a = 8.5223(3) Å, b = 8.5642(3) Å, c = 31.1607(5) Å and volume = 2274.34(12) Å^3. R_{wp} = 10.604%. 12 background parameters, asymmetry, profile parameters, sample displacement, spherical harmonics for preferred orientation and unit cell parameters were refined.

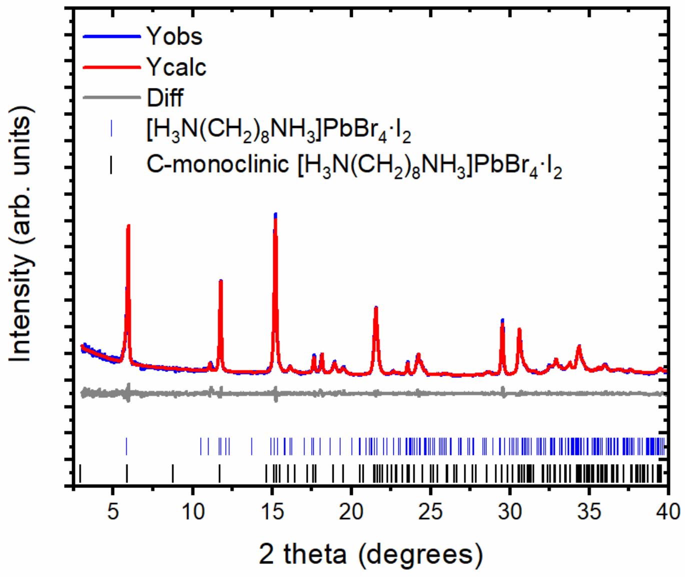

Supplementary Figure 10: Pawley fit for $[H_3N(CH_2)_8NH_3]PbBr_4{\cdot}I_2$. a = 8.416(2) Å, b = 8.366(2) Å, c = 30.392(2) Å, β = 93.1(4)°, volume = 2136.9(8) Å^3. R_{wp} = 6.538 %. A second, C-centred monoclinic cell has been added as a second phase (space group C2), which was fitted using a = 8.289(9) Å, b =8.274(11) Å, c = 30.25(4) Å, β = 92.89(7) °. 12 background parameters, asymmetry, profile parameters, sample displacement and unit cell parameters were refined.

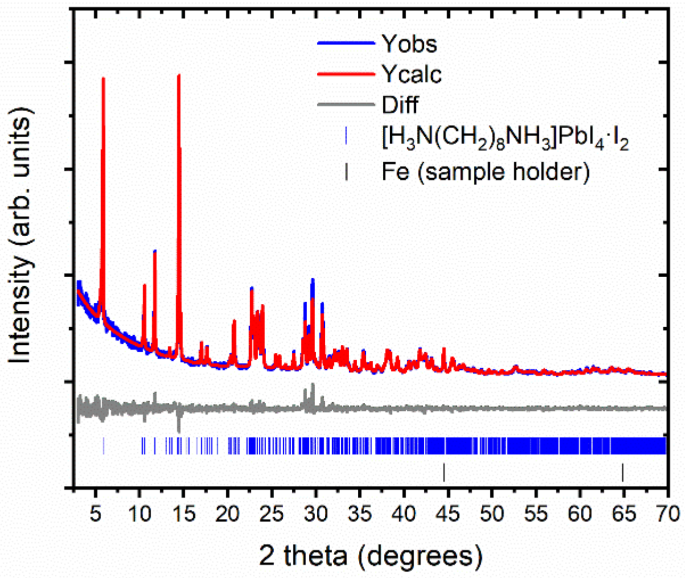

Supplementary Figure 11: Rietveld fit for $[H_3N(CH_2)_8NH_3]PbI_4{\cdot}I_2$. a = 8.8161 (3) Å b = 8.7167(3) Å, c = 30.1314(11), β= 92.530(4)° volume= 2313.28(16) Å^3. R_{wp} =6.647% . 12 background parameters, a term for peak asymmetry, profile parameters, sample displacement, unit cell parameters, atomic coordinates and individual U_{isos} for Pb and I were refined.

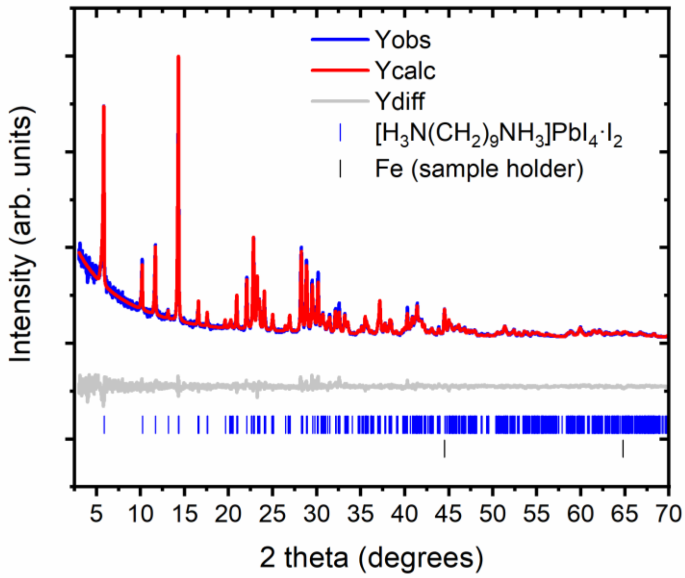

Supplementary Figure 12: Rietveld fit for $[H_3N(CH_2)_9NH_3]PbI_4{\cdot}I_2$. a = 30.2366(10) Å, b = 9.0411(4) Å, c = 8.8124(4) Å, β = 89.576(4)°, volume = 2409.01(16) Å^3, R_{wp} = 6.687%. 12 background parameters, a term for peak asymmetry, profile parameters, sample displacement, unit cell parameters atomic coordinates and individual U_{isos} for Pb and I were refined.

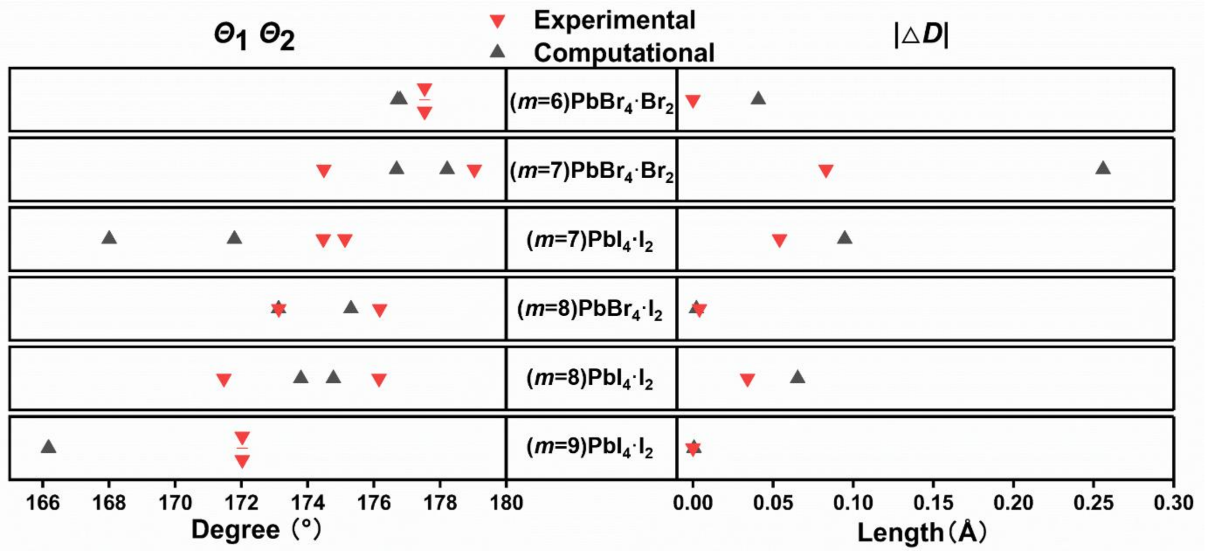

Supplementary Figure 13: Comparisons between computational and experimental values obtained for the key structural parameters: θ_1, θ_2 (scatter with reference to left x-axis) and $|\triangle D|$ (scatter with reference to right x-axis).

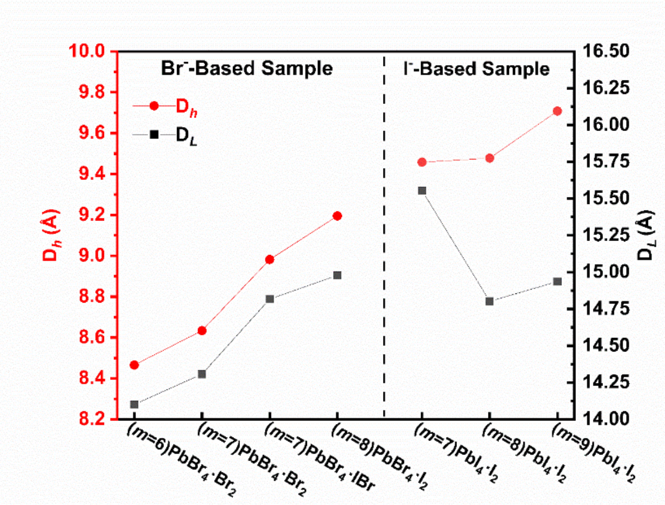

Supplementary Figure 14: Structural parameters D_h (scatter with reference to left y-axis) and D_L (scatter with reference to right y-axis,) for seven intercalated perovskites from analysis of single crystal XRD data.

Supplementary Table 5: Comparison of individual octahedral distortions and inter-octahedral distortions in the $[H_3N(CH_2)_mNH_3]PbX_4$ and $[H_3N(CH_2)_mNH_3]PbX_4{\cdot}X_2$ perovskites, where m = 6-9 and X = Br or I

	Octahedral Distortions		Inter-octahedral Distortions			
Chemical Composition	Δd ($\times 10^{-6}$)	σ^2	Pb-*X*-Pb bond angle (°)	Pb – Pb Distance (Å)	Temperature (K)	Ref
$[H_3N(CH_2)_6NH_3]PbBr_4$	55.63	6.13	149.62	5.84	238	[7]
$[H_3N(CH_2)_6NH_3]PbBr_4{\cdot}Br_2$	0.47	3.43	146.26	5.68	173	[7]
$[H_3N(CH_2)_7NH_3]PbBr_4{\cdot}Br_2$	29.37	8.15	149.20<br>149.75	5.76<br>5.77	173	This work
$[H_3N(CH_2)_7NH_3]PbBr_4{\cdot}IBr$	0.12	2.63	148.21	5.72	298	This work
$[H_3N(CH_2)_7NH_3]PbI_4{\cdot}I_2$	6.11	4.64	144.05<br>146.32	6.03<br>6.05	298	This work
$[H_3N(CH_2)_8NH_3]PbBr_4$	0.62	3.59	148.21	5.75	298	[17]
$[H_3N(CH_2)_8NH_3]PbBr_4{\cdot}I_2$	35.54	17.86	153.39<br>153.76	5.84<br>5.87	173	This work
$[H_3N(CH_2)_8NH_3]PbI_4$	4.21	7.69	147.62	6.16	293	[18]
$[H_3N(CH_2)_8NH_3]PbI_4{\cdot}I_2$	17.48	2.18	151.49<br>146.72	6.15<br>6.09	173	This work
$[H_3N(CH_2)_9NH_3]PbI_4$	34.54	4.33	154.24<br>153.26	6.20<br>6.21	293	[8]
$[H_3N(CH_2)_9NH_3]PbI_4{\cdot}I_2$	5.15	5.16	161.87	6.31	298	This work

$$\boldsymbol{\Delta d} = \left(\frac{1}{6}\right)\sum\left[\frac{d_n - d}{d}\right]^2 \quad \text{Equation (S1)}$$

$$\boldsymbol{\sigma^2} = \sum_{i=1}^{12}\frac{(\theta_i - 90)^2}{11} \quad \text{Equation (S2)}$$

Bond length distortions (**Δd**, Equation S1)[5] and bond angle variances (**σ^2**, Equation S2)[6] were used to describe the distortion of individual octahedra (Supplementary Table 4). In Equation S1, *d* is the average Pb-*X* bond distance and d_n are the six individual bond distances. In Equation S2, **θ_i** is the individual *X*-Pb-*X* angle. No significant Pb-*X* bond length distortion was observed (**Δd** ~10^{-6}, Figure 3a) either before or after intercalation, unlike the (110)-orientated layered perovskites.[7-10] This suggests that the formation of halogen bonds is unlikely to change the Pb-*X* bond length in the axial direction.

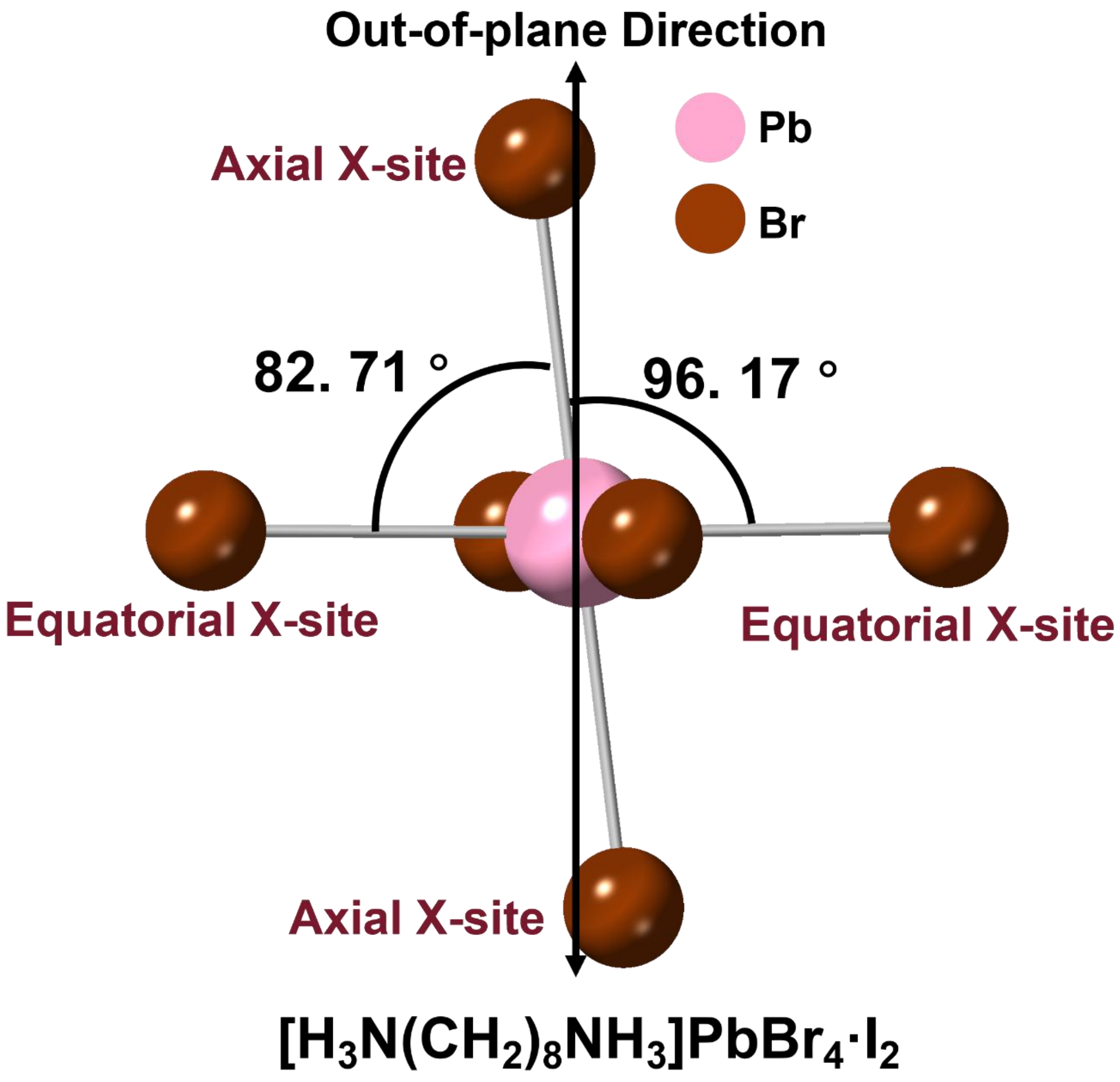

Supplementary Figure 15: Individual $[PbBr_4]^{2-}$ octahedra of $[H_3N(CH_2)_8NH_3]PbBr_4{\cdot}I_2$. The axial Pb-Br bonds tilt in opposite directions to accommodate the intercalation of I_2 molecules.

Interestingly, $[H_3N(CH_2)_8NH_3]PbBr_4{\cdot}I_2$ is the only example which shows a considerable increase in both **Δ*d*** and $\boldsymbol{\sigma^2}$, which is caused by the out-of-plane tilting of inorganic sheets. In this case, the two axial Pb-Br bonds of each octahedra tilt in the opposite directions, so that all $Br_{(axial)}$-Pb-$Br_{(equatorial)}$ bonds are in the range 82.7° - 96.2,° but the $Br_{(axial)}$-Pb- $Br_{(axial)}$ bond is still close to the ideal 180° (179.8°), as shown in Supplementary Figure 15.

From the comparison of *m* =7 intercalated perovskites (Figure 3b), $H_3N(CH_2)_7NH_3]PbI_4{\cdot}I_2$ has the lowest **Δ*d*** and $\boldsymbol{\sigma^2}$ values. This agrees with octahedral distortion being smaller when the layer shift is smaller (Figure 2b), as discussed previously. [11] $[H_3N(CH_2)_7NH_3]PbBr_4{\cdot}Br_2$, has the highest values, as its $[H_3N(CH_2)_7NH_3]^{2+}$ cation adopts a larger cross-sectional area than those ‘all-*trans*’ or elongated carbon chains found in $[H_3N(CH_2)_7NH_3]PbBr_4{\cdot}IBr$ and $[H_3N(CH_2)_7NH_3]PbI_4{\cdot}I_2$, respectively.

Supplementary Discussion, Tables and Figures: Thermal analysis and Diffuse Reflectance UV-Visible Spectroscopy

Thermal Analysis

In order to assess the thermal stability of the intercalated samples, thermogravimetric analysis (TGA) was carried out on $[H_3N(CH_2)_mNH_3]PbX_4{\cdot}X_2$ and $[H_3N(CH_2)_mNH_3]PbX_4{\cdot}XX$ (see Supplementary Table 5 and Supplementary Figure 16). Br_2 deintercalates at lower temperatures than I_2 across the range of compounds. The deintercalation of IBr from $[H_3N(CH_2)_7NH_3]PbBr_4{\cdot}IBr$ occurs at temperatures intermediate to $[H_3N(CH_2)_7NH_3]PbBr_4{\cdot}Br_2$ and $[H_3N(CH_2)_7NH_3]PbI_4{\cdot}I_2$. Of the samples studied here, $[H_3N(CH_2)_7NH_3]PbBr_4{\cdot}Br_2$ is the least thermally stable, whilst $[H_3N(CH_2)_7NH_3]PbI_4{\cdot}I_2$ is the most.

Supplementary Table 6: Results from Thermogravimetric Analysis carried out on $[H_3N(CH_2)_mNH_3]PbX_4{\cdot}XX$ where m = 7-9 and X = Br or I. The expected mass loss is calculated assuming the loss of one halogen molecule per formula unit.

Sample	Expected Mass Lost (%)	Mass Lost at 250 ℃ (%)
$[H_3N(CH_2)_7NH_3]PbBr_4{\cdot}Br_2$	19.52	19.77
$[H_3N(CH_2)_7NH_3]PbBr_4{\cdot}IBr$	23.88	24.40
$[H_3N(CH_2)_7NH_3]PbI_4{\cdot}I_2$	23.06	23.01
$[H_3N(CH_2)_8NH_3]PbBr_4{\cdot}I_2$	27.38	26.31
$[H_3N(CH_2)_8NH_3]PbI_4{\cdot}I_2$	22.77	21.42
$[H_3N(CH_2)_9NH_3]PbI_4{\cdot}I_2$	22.48	20.34

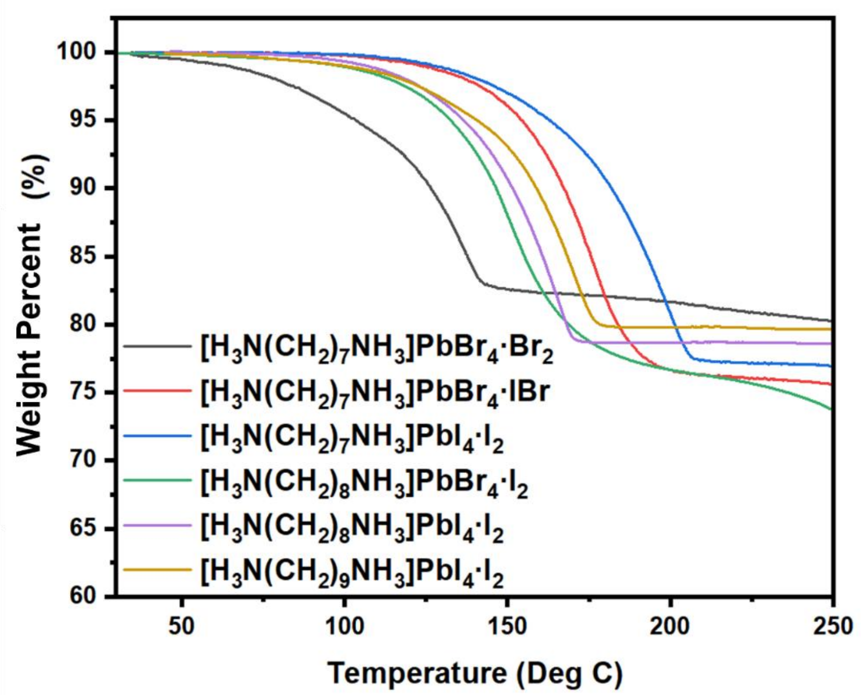

Supplementary Figure 16: Thermogravimetric analysis of $[H_3N(CH_2)_mNH_3]PbX_4{\cdot}XX$ where m = 7-9 and X = Br or I. TGA was carried out on single crystal samples using a heating rate of 5 °C min^{-1} in the temperature range 25- 250 °C, under air.

Diffuse Reflectance UV-Visible Spectroscopy

Diffuse Reflectance UV-Visible Spectroscopy was carried out in order to assess the optical band gap of the perovskites (Supplementary Figure 17 and Supplementary Table 6). $[H_3N(CH_2)_7NH_3]PbI_4{\cdot}I_2$ had the lowest optical band gap (1.77 eV), whilst $[H_3N(CH_2)_7NH_3]PbBr_4{\cdot}Br_2$ had the highest optical band gap (2.39 eV).

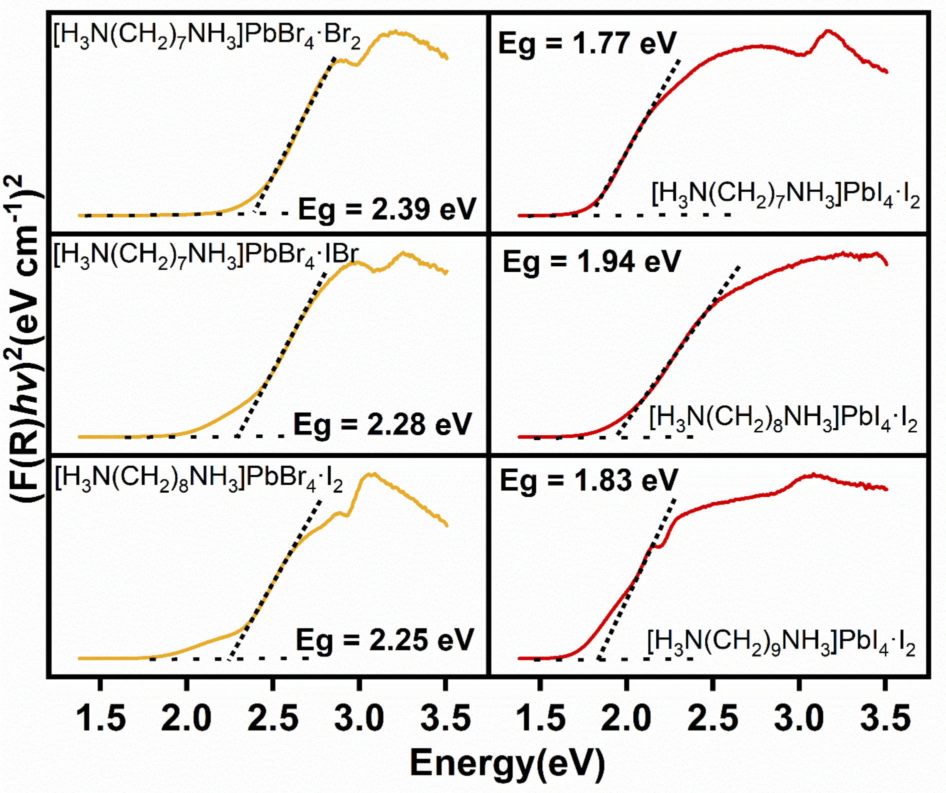

Supplementary Figure 17: Diffuse reflectance spectra of $[H_3N(CH_2)_mNH_3]PbX_4{\cdot}XX$ where m = 7-9 and X = Br or I plotted using the Kubelka Munk transformation.

Supplementary Table 7: Optical band gaps determined using UV-visible diffuse reflectance spectroscopy for new intercalated perovskites, $[H_3N(CH_2)_mNH_3]PbX_4{\cdot}X_2$ and $[H_3N(CH_2)_mNH_3]PbX_4{\cdot}XX$ where m = 7-9 and X = Br or I.

Sample	Band Gap (eV)
$[H_3N(CH_2)_7NH_3]PbBr_4{\cdot}Br_2$	**2.39**
$[H_3N(CH_2)_7NH_3]PbBr_4{\cdot}IBr$	**2.28**
$[H_3N(CH_2)_7NH_3]PbI_4{\cdot}I_2$	**1.77**
$[H_3N(CH_2)_8NH_3]PbBr_4{\cdot}I_2$	**2.25**
$[H_3N(CH_2)_8NH_3]PbI_4{\cdot}I_2$	**1.94**
$[H_3N(CH_2)_9NH_3]PbI_4{\cdot}I_2$	**1.83**

Supplementary Discussion and Figures: Thin Film Fabrication

Discussion of Thin Film Fabrication

The initial synthesis of $[H_3N(CH_2)_8NH_3]PbI_4{\cdot}I_2$ was attempted via spin coating to assess the practicality of fabricating thin films containing intercalated halogen molecules. We initially attempted this by directly spin-coating a precursor solution containing excess I_2. However, the extra halogen is thought to weaken the surface tension of the solution, [12,13,] which also accelerates the nucleation speed of the intercalated perovskite and hence contributes to the poor surface coverage of the thin film (Figure 4B, main text).[14]

A second film-fabrication method was tested, which involves the post-synthetic intercalation of I_2 into $[H_3N(CH_2)_8NH_3]PbI_4$ films (see Figure 4e, 4f, main text). The surface coverage and uniformity of the films significantly improved when the precursor solutions were stoichiometric, rather than containing excess halogen.[14,15]

Although $[H_3N(CH_2)_6NH_3]PbI_4{\cdot}Br_2$ and $[H_3N(CH_2)_7NH_3]PbI_4{\cdot}Br_2$ were predicted to be the best photovoltaic materials based on our computational studies, we were unable to synthesise these materials in any bulk form (either single crystals or polycrystalline samples), due to halide exchange between $[PbX_4]_\infty$ layers and intercalated halogen molecules. Karunadasa has found that halide exchange in 3D perovskites is chemically favoured. [16]

Film fabrication of $[H_3N(CH_2)_6NH_3]PbI_{4\text{-}y}Br_y{\cdot}xBr_2$

$[H_3N(CH_2)_6NH_3]PbI_4{\cdot}Br_2$ can only be prepared in thin-film form by the intercalation of Br_2 into a film of $[H_3N(CH_2)_6NH_3]PbI_4$, but the exact amount of Br_2 intercalated (or halide-exchanged into the lead iodide layers) could not be quantified, hence this sample was denoted as $[H_3N(CH_2)_6NH_3]PbI_{4\text{-}y}Br_y{\cdot}xBr_2$. The sample was prepared by controlling the amount of Br_2 through two different methods: 1) dry-ice cooling (Supplementary Figure 18) and 2) thin-film transfer (Supplementary Figure 19).

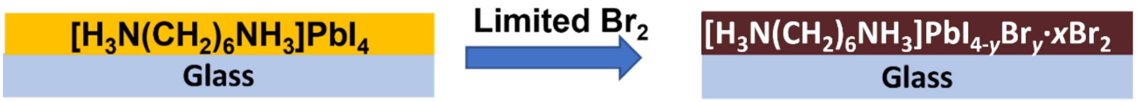

Supplementary Figure 18: Method 1 for preparation of $[H_3N(CH_2)_6NH_3]PbI_{4\text{-}y}Br_y{\cdot}xBr_2$, controlling the amount of Br_2 introduced to a film of $[H_3N(CH_2)_6NH_3]PbI_4$ by dry-ice cooling.

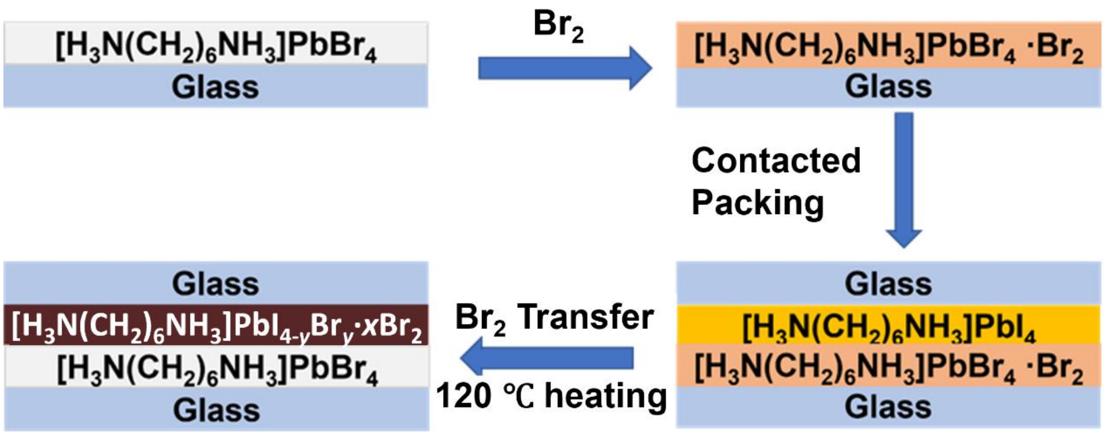

Supplementary Figure 19: Method 2 for preparation of $[H_3N(CH_2)_6NH_3]PbI_{4-y}Br_y{\cdot}xBr_2$, using $[H_3N(CH_2)_6NH_3]PbBr_4{\cdot}Br_2$ to transfer a limited amount of Br_2 to a film $[H_3N(CH_2)_6NH_3]PbI_4$ by direct contact of the two films.

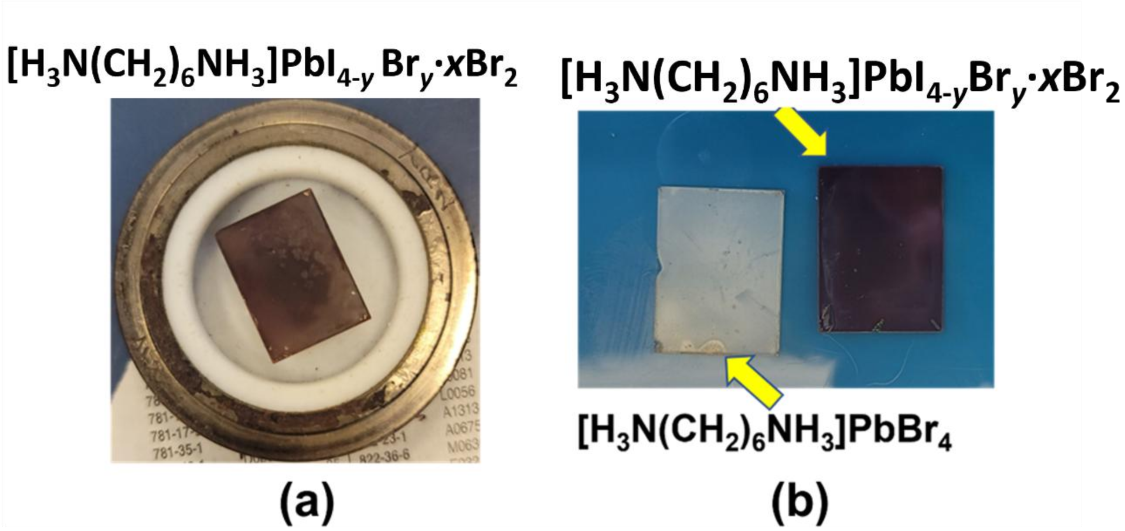

Supplementary Figure 20: Photographs of films resulting from (a) dry-iced cooling (method 1) and (b) the thin-film transfer (method 2).

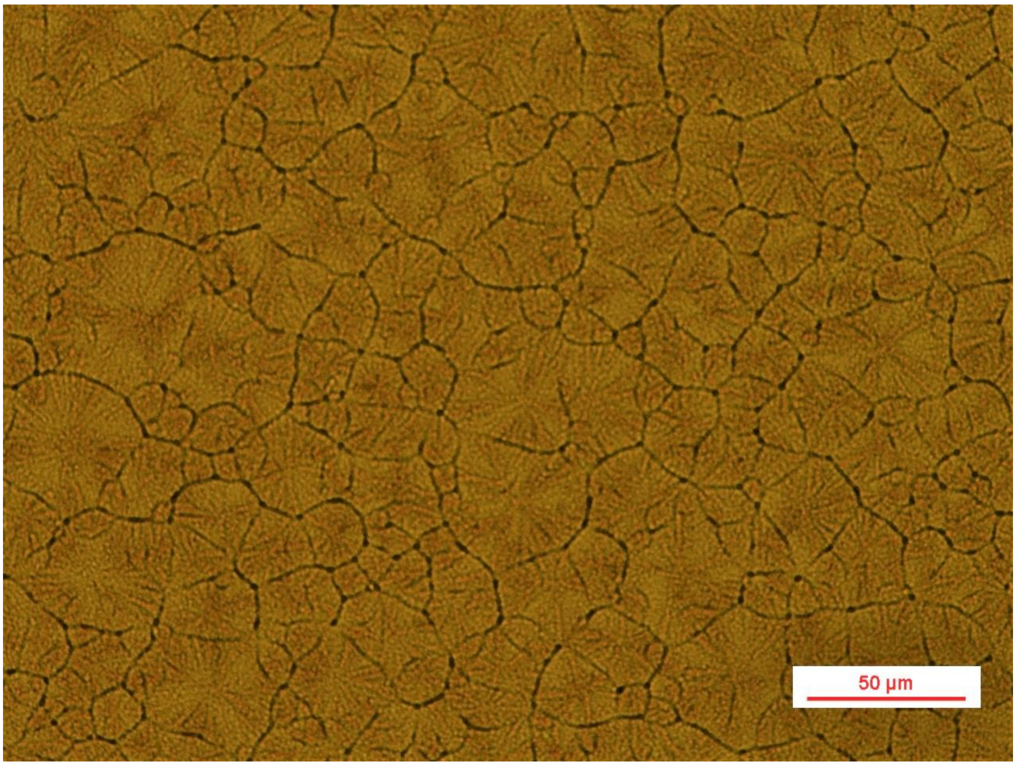

Supplementary Figure 21: Optical microscope image of $[H_3N(CH_2)_8NH_3]PbI_4$ after deintercalation by heating $[H_3N(CH_2)_8NH_3]PbI_4{\cdot}I_2$ at 80 °C on a hotplate for 5 mins.

Supplementary Table 8: Summary of the computational and experimental agreement for the ten intercalated perovskites $[H_3N(CH_2)_mNH_3]PbX_4{\cdot}X_2$ and $[H_3N(CH_2)_mNH_3]PbX_4{\cdot}XX$

Chemical Formula	Computational Prediction	Single Crystal Sample	Thin Film (Post-Synthetic)
$[H_3N(CH_2)_6NH_3]PbBr_4{\cdot}Br_2$	Accessible	Accessible	Accessible
$[H_3N(CH_2)_6NH_3]PbI_4{\cdot}Br_2$	Accessible	Non-Accessible	Accessible
$[H_3N(CH_2)_7NH_3]PbBr_4{\cdot}Br_2$	Accessible	Accessible	Non-Accessible
$[H_3N(CH_2)_7NH_3]PbBr_4{\cdot}IBr$	Not Included	Accessible	Non-Accessible
$[H_3N(CH_2)_7NH_3]PbI_4{\cdot}Br_2$	Accessible	Non-Accessible	Non-Accessible
$[H_3N(CH_2)_7NH_3]PbI_4{\cdot}I_2$	Accessible	Accessible	Non-Accessible
$[H_3N(CH_2)_8NH_3]PbBr_4{\cdot}Br_2$	Accessible	Low-Quality	Accessible
$[H_3N(CH_2)_8NH_3]PbBr_4{\cdot}I_2$	Accessible	Accessible	Non-Accessible
$[H_3N(CH_2)_8NH_3]PbI_4{\cdot}I_2$	Accessible	Accessible	Accessible
$[H_3N(CH_2)_9NH_3]PbI_4{\cdot}I_2$	Accessible	Accessible	Accessible

Raman Spectra of Thin Film Samples

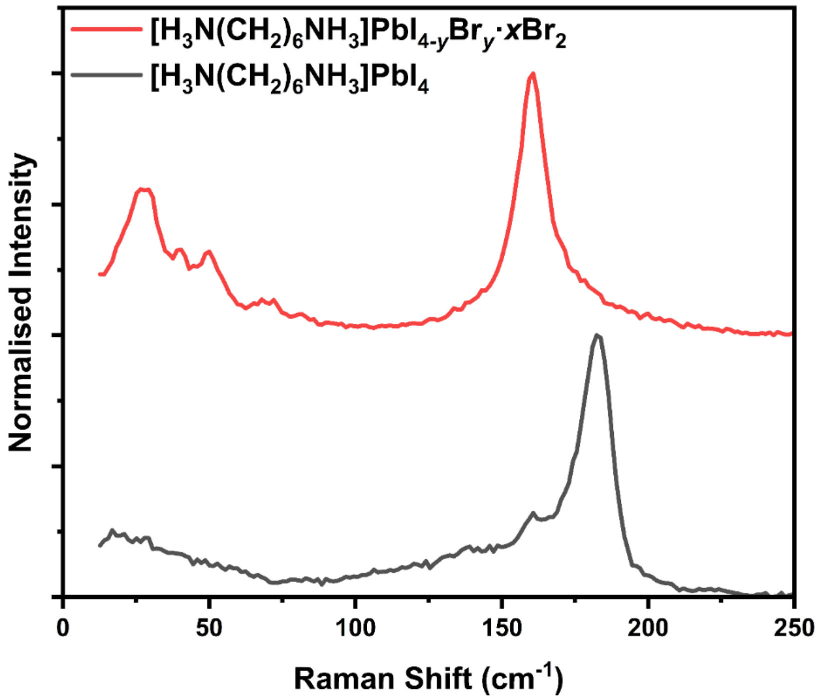

Supplementary Figure 22: Raman spectra of $[H_3N(CH_2)_6NH_3]PbI_4$ and $[H_3N(CH_2)_6NH_3]PbI_{4-y}Br_y{\cdot}xBr_2$

We were unable to confirm the precise composition of the $[H_3N(CH_2)_6NH_3]PbI_4{\cdot}Br_2$ film so this sample was denoted as $[H_3N(CH_2)_6NH_3]PbI_{4-y}Br_y{\cdot}xBr_2$ and the low stability of Br_2 in this film precluded the use of PXRD to check the phase purity of the sample. Hence low wavenumber Raman

spectroscopy (Supplementary Figure 22) was used to compare $[H_3N(CH_2)_6NH_3]PbI_4$ and $[H_3N(CH_2)_6NH_3]PbI_{4-y}Br_y{\cdot}xBr_2$ samples. Supplementary Figure 23 shows the low wavenumber Raman spectra of $[H_3N(CH_2)_8\ NH_3]PbI_4$ and $[H_3N(CH_2)_8\ NH_3]PbI_4{\cdot}I_2$.

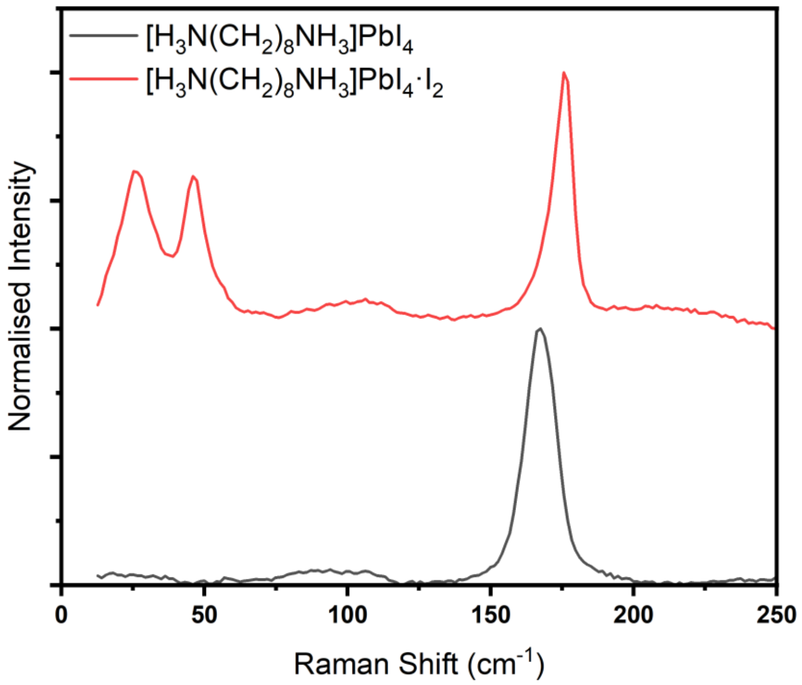

Supplementary Figure 23: Raman spectra of $[H_3N(CH_2)_8NH_3]PbI_4$ and $[H_3N(CH_2)_8NH_3]PbI_4{\cdot}I_2$

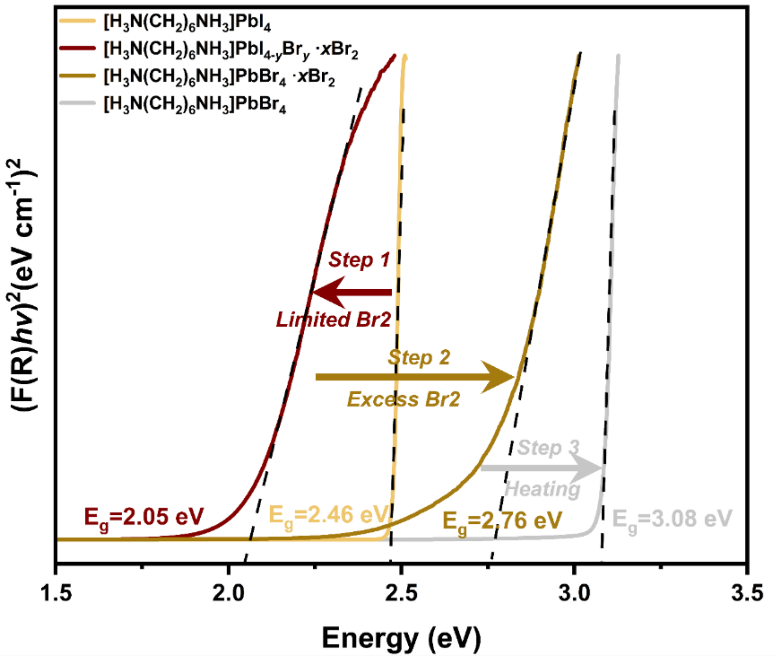

Supplementary Figure 24: UV-visible diffuse reflectance spectra of thin-films of four layered perovskites $[H_3N(CH_2)_6NH_3]PbI_4$, $[H_3N(CH_2)_6NH_3]PbBr_4$, $[H_3N(CH_2)_6NH_3]PbI_{4-y}Br_y{\cdot}xBr_2$ and $[H_3N(CH_2)_6NH_3]PbBr_4{\cdot}Br_2$. The intercalated perovskites, $[H_3N(CH_2)_6NH_3]PbI_{4-y}Br_y{\cdot}xBr_2$ and $[H_3N(CH_2)_6NH_3]PbBr_4{\cdot}Br_2$ were prepared using the post-synthetic Br_2 intercalation method.

In order to provide further evidence for the intercalation of Br_2 in $[H_3N(CH_2)_6NH_3]PbI_4$, we carried out diffuse reflectance UV-Vis spectroscopy, firstly for a thin film of $[H_3N(CH_2)_6NH_3]PbI_4$. The film was then exposed to ice-cold Br_2. We note that ice was used in this step, rather than dry ice, due to dry ice shortages, but the rest of the experimental procedures remained the same as the method used for fabrication of $[H_3N(CH_2)_6NH_3]PbI_4{\cdot}Br_2$ thin films.

As shown in Supplementary Figure 24, the original $[H_3N(CH_2)_6NH_3]PbI_4$ film has a bandgap 2.46 eV, in agreement with the value of 2.44 eV reported by others.[31] Once the $[H_3N(CH_2)_6NH_3]PbI_4$ film was put into contact with a limited amount of Br_2 (Step 1), the colour changed to a deep red colour and the band gap dropped to 2.05 eV. The composition of this film has been denoted as $[H_3N(CH_2)_6NH_3]PbI_{4-y}Br_y{\cdot}xBr_2$. The band gap obtained for this sample is lower than both the pure $[H_3N(CH_2)_6NH_3]PbBr_4{\cdot}Br_2$ we reported previously (2.15 eV) [11] and $[H_3N(CH_2)_6NH_3]PbI_4$. This provides some evidence to show that intercalated bromine molecules and *X*-site iodide ions co-exist in the sample ($y < 4$ and $x > 0$). The $[H_3N(CH_2)_6NH_3]PbI_{4-y}Br_y{\cdot}xBr_2$ film was then exposed to an excess of Br_2 (Step 2), until its colour reverted to yellow. The band gap of the resulting film increased to 2.76 eV and this band gap is higher than the band gap of 2.15 eV expected for $[H_3N(CH_2)_6NH_3]PbBr_4{\cdot}Br_2$ and lower than the 3.08 eV expected for $[H_3N(CH_2)_6NH_3]PbBr_4$, which suggests that the complete intercalation of Br_2 has not occurred, so the sample composition can be thought of as $[H_3N(CH_2)_6NH_3]PbBr_4{\cdot}xBr_2$ ($x < 1$). This film has low thermal stability and its colour visibly lightens on standing at room temperature. If heated to 60 °C for 2 minutes (Step 3), the Br_2 can be fully

deintercalated, leaving a white $[H_3N(CH_2)_6NH_3]PbBr_4$ film with a bandgap of 3.08 eV. PXRD was used to check the purity of the initial $[H_3N(CH_2)_6NH_3]PbI_4$ film and resulting $[H_3N(CH_2)_6NH_3]PbBr_4$ film (Supplementary Figure 25 and 26).

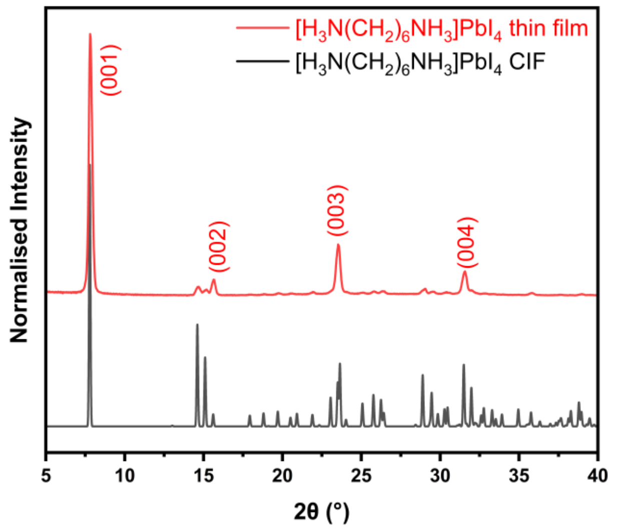

Supplementary Figure 25: PXRD pattern of the $[H_3N(CH_2)_6NH_3]PbI_4$ thin film sample, compared with the simulated pattern of bulk$[H_3N(CH_2)_6NH_3]PbI_4$.[17]

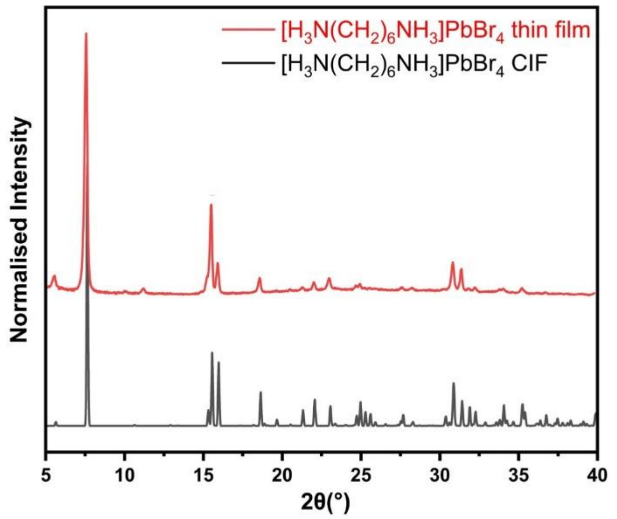

Supplementary Figure 26: PXRD pattern of $[H_3N(CH_2)_6NH_3]PbBr_4$ thin film sample, compared with the simulated pattern of bulk $[H_3N(CH_2)_6NH_3]PbBr_4$.[11]

We noticed that the competition between halide ion exchange (*i.e.* the formation of $[H_3N(CH_2)_6NH_3]PbBr_{4-y}I_y$) and halogen intercalation (*i.e.* formation of $[H_3N(CH_2)_mNH_3]PbX_4{\cdot}xBr_2$) is highly dependent on the reaction temperature. In the post-synthetic intercalation procedure, which utilises a dry ice-cooling step, a clear gap between Step 1 and Step 2 can be found. As a result, the $[H_3N(CH_2)_6NH_3]PbI_{4-y}Br_y{\cdot}xBr_2$ film obtained in Step 1 using dry-ice is much darker in colour than the film prepared using ice, even though the exposure times are the same. Halide ion substitution (*i.e.* increasing the value of y in $[H_3N(CH_2)_6NH_3]PbI_{4-y}Br_y{\cdot}xBr_2$) is slower at lower temperature, which provides some initial observations that it may be possible to prepare pure $[H_3N(CH_2)_6NH_3]PbI_4{\cdot}Br_2$ compounds at low temperature. Due to time constraints, we were unable to obtain the band gaps of $[H_3N(CH_2)_6NH_3]PbI_{4-y}Br_y$ films prepared at different temperatures.

Supplementary Discussion and Figures: Photoluminescence

Discussion of photoluminescence results

We note that considering the spatial arrangement of molecular orbitals in real space, excitation from the *X*-site anion *p* orbitals to lead *p* orbitals allow electrons to move into the halogen bond. Therefore, exciting the intercalated band individually is very weak. We used a green constant wavelength laser (530 nm) to activate $[H_3N(CH_2)_8NH_3]PbI_4{\cdot}I_2$ at 4 K and no broadband emission was observed. This is in contrast to what was observed using the 415 nm laser, *vide infra*. However, when using a high energy tuneable pulsed laser, weak emission from the broadband was observed with excitation up to 540 nm. The pulsed laser was used to measure photoluminescence excitation spectra discussed in the main text. Supplementary Figure 27-30 show the results from PL experiments using the 415 nm laser.

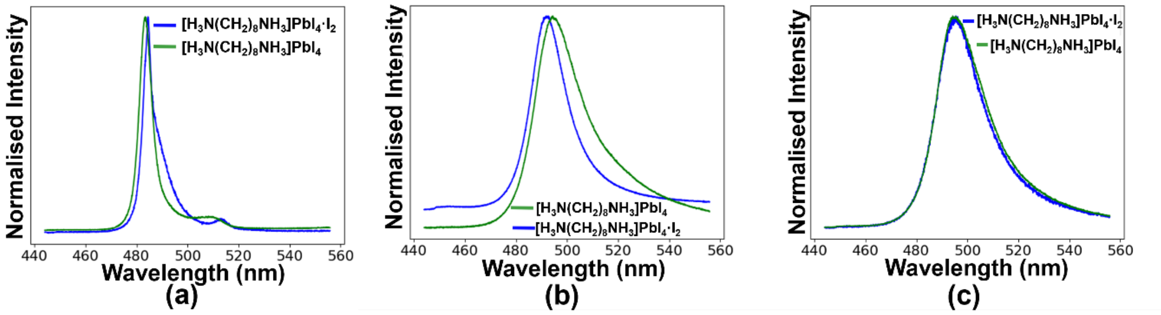

Supplementary Figure 27: Narrowband photoluminescence of (a) $[H_3N(CH_2)_8NH_3]PbI_4$ and $[H_3N(CH_2)_8NH_3]PbI_4{\cdot}I_2$ crystals at 4K, (b) $[H_3N(CH_2)_8NH_3]PbI_4$ and $[H_3N(CH_2)_8NH_3]PbI_4{\cdot}I_2$ crystals at room temperature, and (c) $[H_3N(CH_2)_8NH_3]PbI_4$ and $[H_3N(CH_2)_8NH_3]PbI_4{\cdot}I_2$ thin films at room temperature.

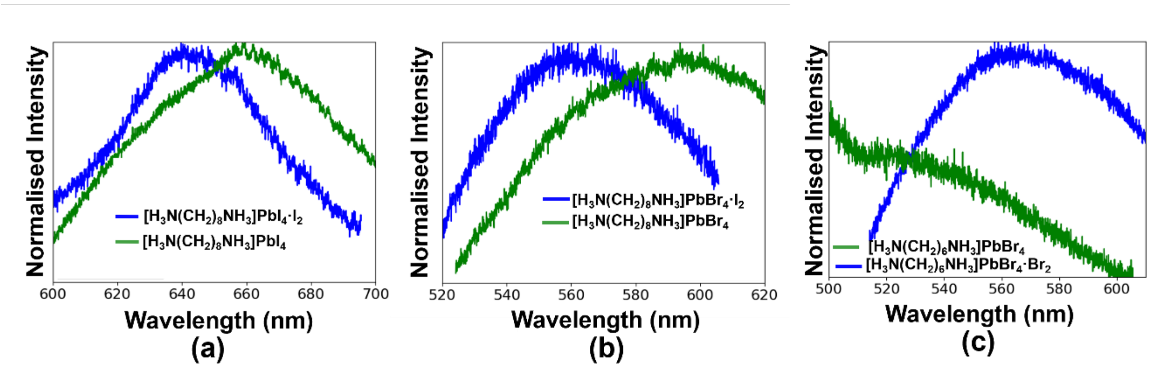

Supplementary Figure 28: Broadband photoluminescence of (a) $[H_3N(CH_2)_8NH_3]PbI_4$ and $[H_3N(CH_2)_8NH_3]PbI_4{\cdot}I_2$ crystals at 4K; (b) $[H_3N(CH_2)_8NH_3]PbBr_4$ and $[H_3N(CH_2)_8NH_3]PbBr_4{\cdot}I_2$ Crystal at 4K; $[H_3N(CH_2)_6NH_3]PbBr_4$ and $[H_3N(CH_2)_6NH_3]PbBr_4{\cdot}Br_2$ crystals at 4K

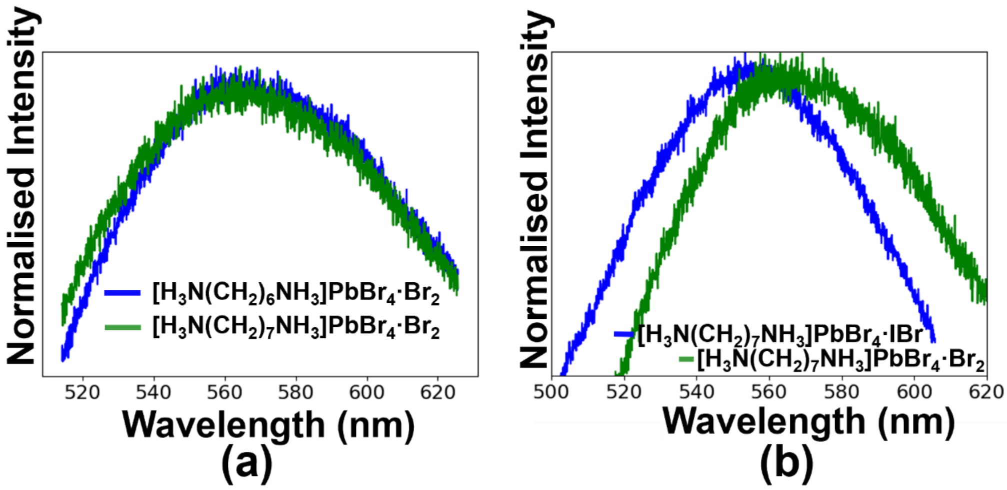

Supplementary Figure 29: (a) Photoluminescence broad peak of $[H_3N(CH_2)_7NH_3]PbBr_4{\cdot}Br_2$ and $[H_3N(CH_2)_6NH_3]PbBr_4{\cdot}Br_2$ crystals at 4K, (b) Photoluminescence broad peak of $[H_3N(CH_2)_7NH_3]PbBr_4{\cdot}Br_2$ and $[H_3N(CH_2)_7NH_3]PbBr_4{\cdot}IBr$ crystals at 4K

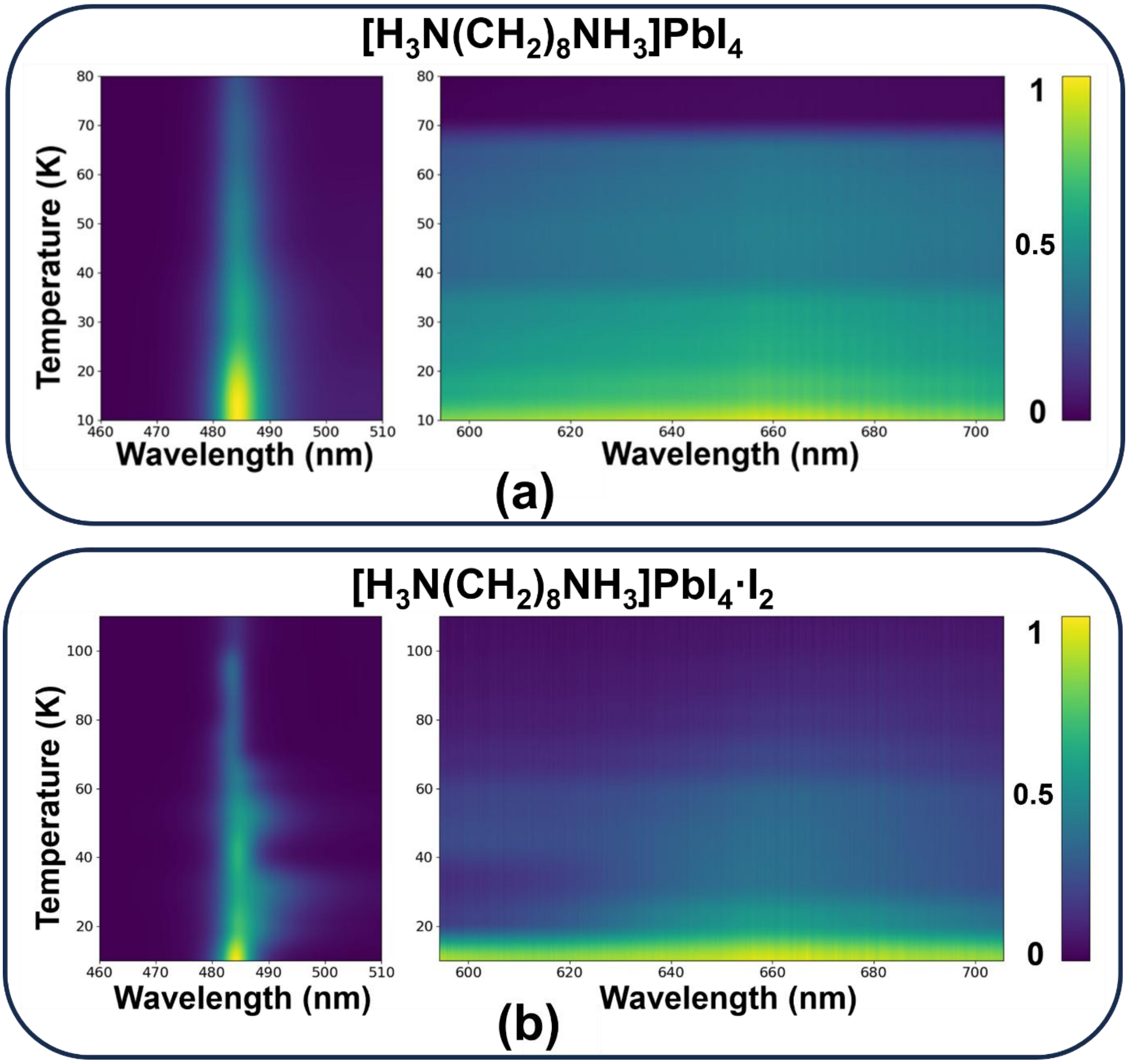

Supplementary Figure 30: (a) Variable temperature photoluminescence of $[H_3N(CH_2)_8NH_3]PbI_4$; (b) Variable temperature photoluminescence of $[H_3N(CH_2)_8NH_3]PbI_4 \cdot I_2$

Supplementary Discussion and Figures: Combined Optical Microscopy and Photoluminescence Experiments

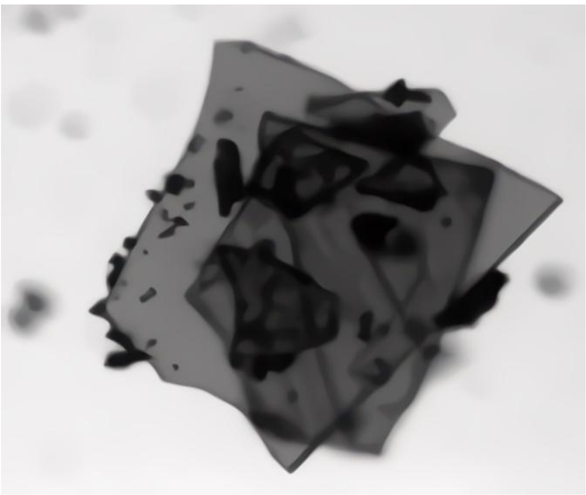

Supplementary Figure 31: Transmission image of the $[H_3N(CH_2)_8NH_3]PbI_4{\cdot}I_2$ crystals using a tungsten light source. A stack of crystals are partially overlapped, have different thicknesses, and are topped by smaller crystallites.

In order to consider the origins of the broadband emissions, we carried out PL experiments for both thin-film form and exfoliated crystal flakes of $[H_3N(CH_2)_8NH_3]PbI_4$ and $[H_3N(CH_2)_8NH_3]PbI_4{\cdot}I_2$ at room temperature. As shown in Supplementary Figure 36, the $[H_3N(CH_2)_8NH_3]PbI_4{\cdot}I_2$ crystal shows sharp emissions under the excitation of UV (425 nm) and blue (515 nm) laser. The excitation from the crystal is uniform, and not influenced by the thickness of the crystals. No excitation is detected under the green light source (590 nm). Additionally, as observed through optical microscopy during excitation (Supplementary Figure 36) the edge of the crystals does not exhibit any 'brighter' excitation than the centre of the crystals, indicating that edge-emission is not observed.

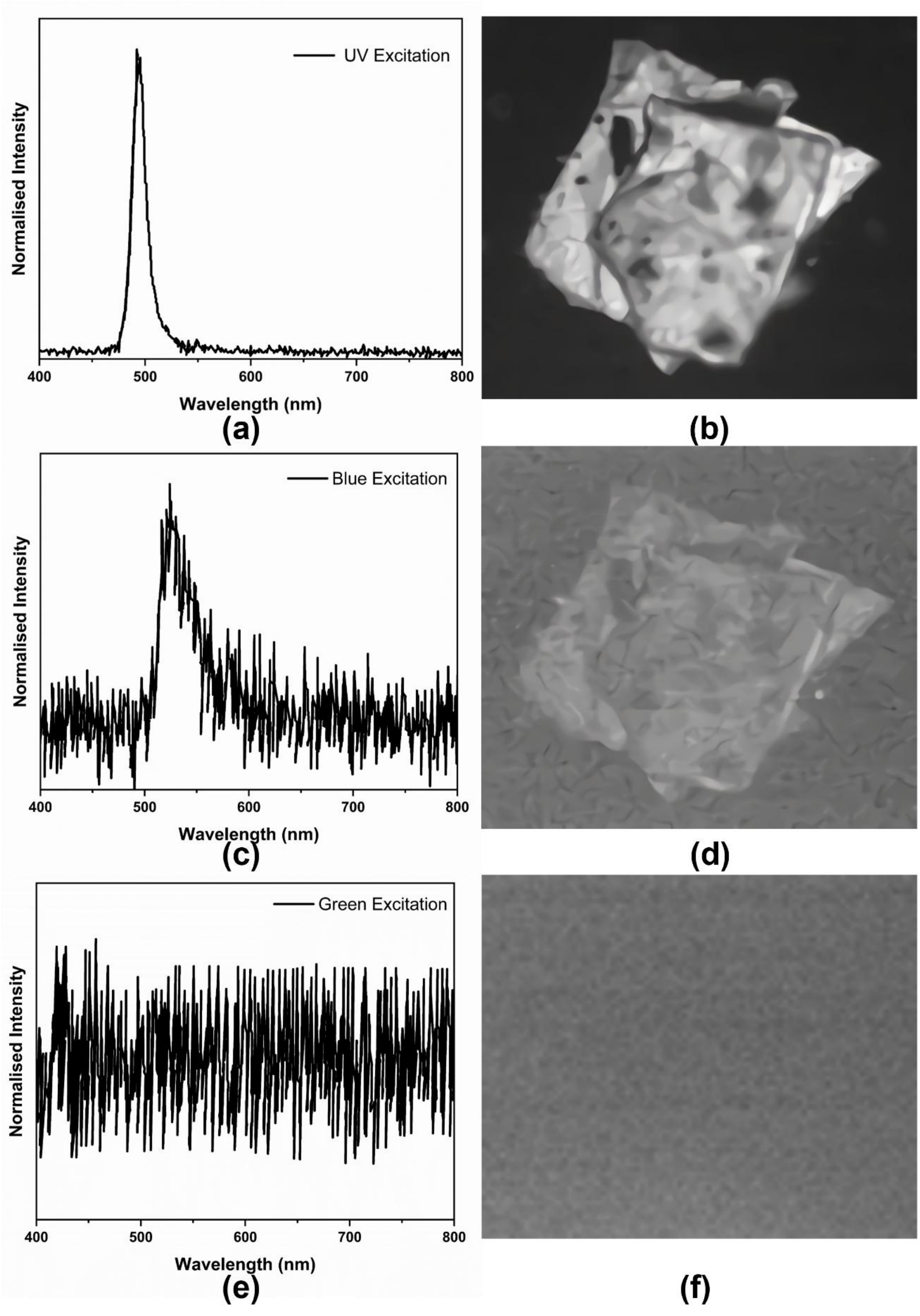

Supplementary Figure 32: Excitation of $[H_3N(CH_2)_8NH_3]PbI_4 \cdot I_2$ crystals by (a) UV laser source 425 nm wavelength; (c) blue laser source 515 nm wavelength and (e) green laser source 590 nm wavelength. (b), (d), (f) Optical micrographs of the crystals under excitation.

Supplementary Discussion and Figures: Photovoltaic Device Fabrication

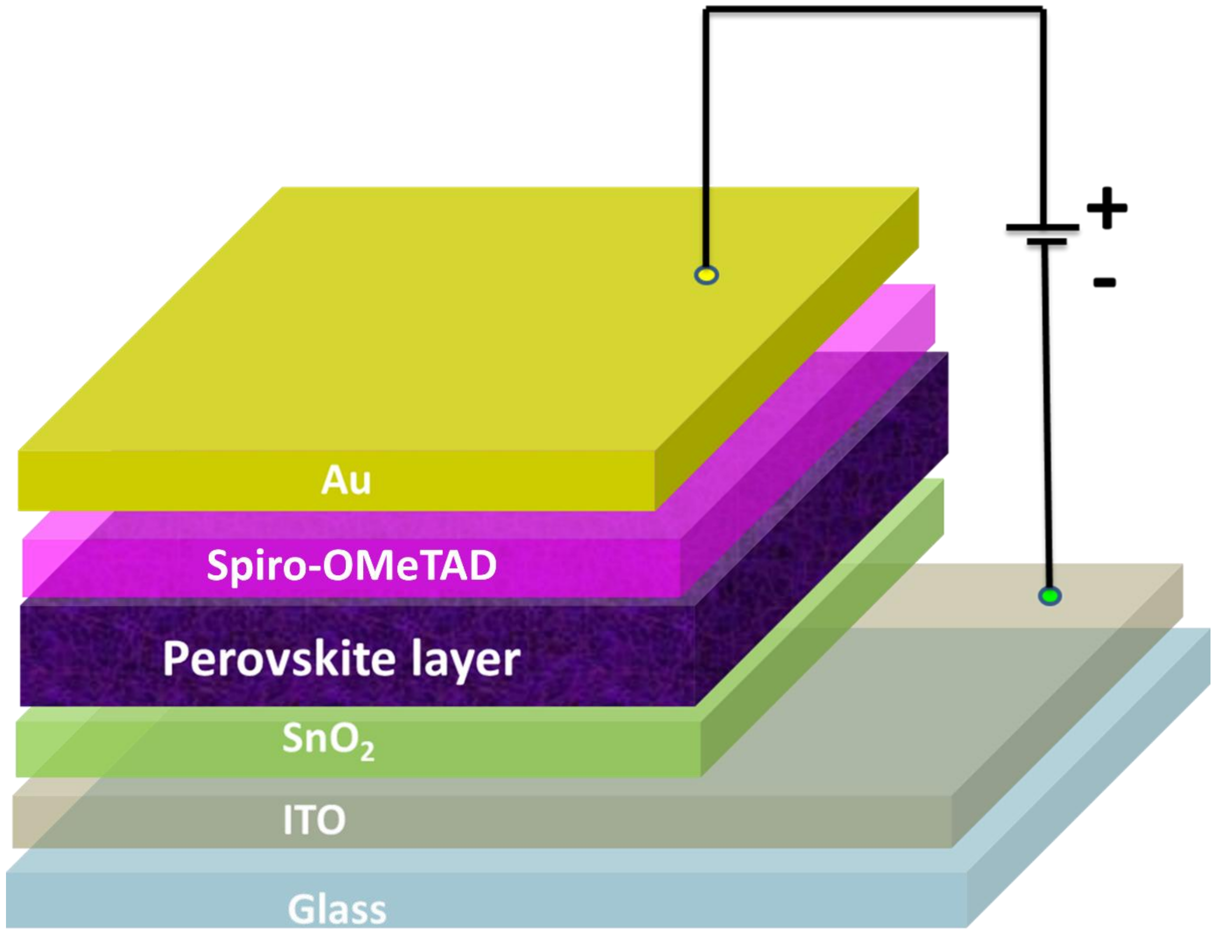

Supplementary Figure 33: n-i-p structure of device tested for the $[H_3N(CH_2)_8NH_3]PbI_4$ and $[H_3N(CH_2)_8NH_3]PbI_4{\cdot}I_2$ perovskites.

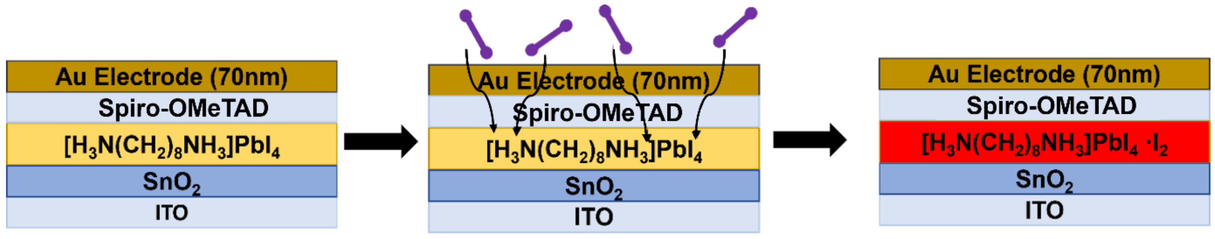

Supplementary Figure 34: Post-synthetic intercalation method for fabrication of $[H_3N(CH_2)_8NH_3]PbI_4{\cdot}I_2$ intercalated perovskite device.

The intercalated perovskite, $[H_3N(CH_2)_8NH_3]PbI_4{\cdot}I_2$ is unstable at the high temperatures required for Au evaporation during photovoltaic device manufacture, so we firstly prepared $[H_3N(CH_2)_8NH_3]PbI_4{\cdot}I_2$ films on the top of SnO_2 layers, expecting that a dense layer of Spiro-OMeTAD would prevent the loss of I_2 during the heating. However, the perovskite layer of the resulting devices which contained spiro-OMeTAD became milky yellow in colour and not the original red colour. A carbon electrode (prepared at 60 °C) was also applied to this device, but I_2 loss was still a problem. Only the post-synthetic method could be successfully used to fabricate devices which

utilised the intercalated $[H_3N(CH_2)_8NH_3]PbI_4{\cdot}I_2$ perovskite (Supplementary Figure 34). The resulting J-V curves are given in Supplementary Figure 35 and S36.

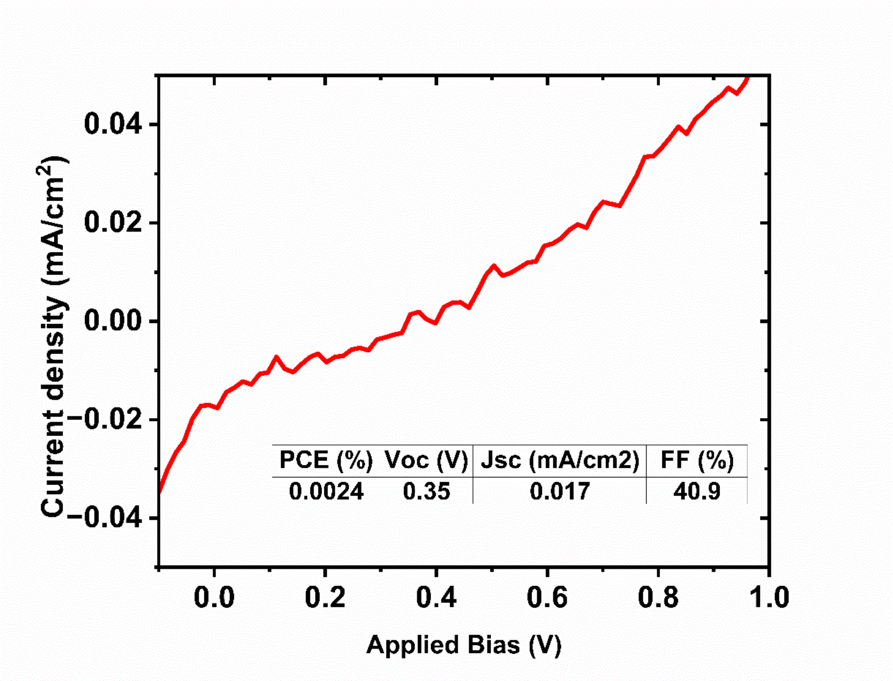

Supplementary Figure 35: J-V curve and photovoltaic performance of the $[H_3N(CH_2)_8NH_3]PbI_4$ based device.

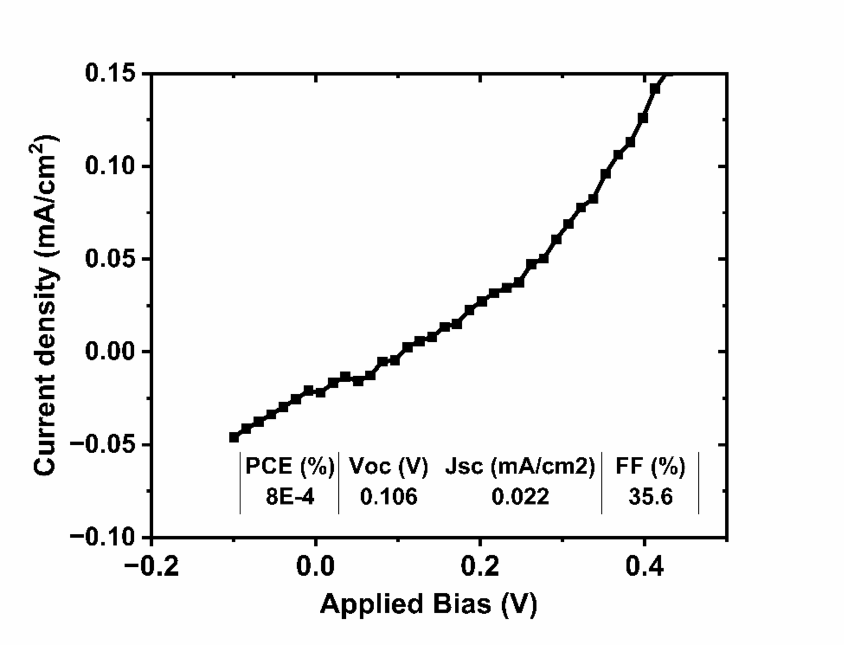

Supplementary Figure 36: J-V curve and photovoltaic performance of $[H_3N(CH_2)_8NH_3]PbI_4{\cdot}I_2$ based device.

From our preliminary photovoltaic device testing, the main observation is that the PCE drops upon intercalation of I_2 into $[H_3N(CH_2)_8NH_3]PbI_4$ and this drop is mainly due to the drop in the V_{oc}. This could be due to the interfacial layer damage of the Spiro-OMeTAD layer due to contact with iodine molecules, as has been observed by others. [18]

Up to the date of submission, we have been unable to develop a good method to fabricate working devices based on $[H_3N(CH_2)_8NH_3]PbI_4{\cdot}I_2$.

Supplementary Discussion and Figures: Layer shift factors (*Ls*) calculation

In order to calculate the layer shift factor, L*s*, the crystal structure should be viewed along the out of plane direction, so that the long stacking axis is pointing towards you. The highest symmetry ideal Ruddlesden-Popper or Dion-Jacobson phases adopt tetragonal symmetry. [19,20,] Many other related structures, including those reported in this paper adopt orthorhombic or monoclinic crystal symmetry, so *Ls* must be calculated using a slightly different method. For orthorhombic structures, as β = 90°, the out-of-plane direction aligns with one of the unit cell axes, which has been denoted as *c* in this example (Supplementary Figure 37). For monoclinic crystal systems, where the $\beta \neq$ 90°, the projection process requires a rotation of (β - 90)° to align the out-of-plane direction.

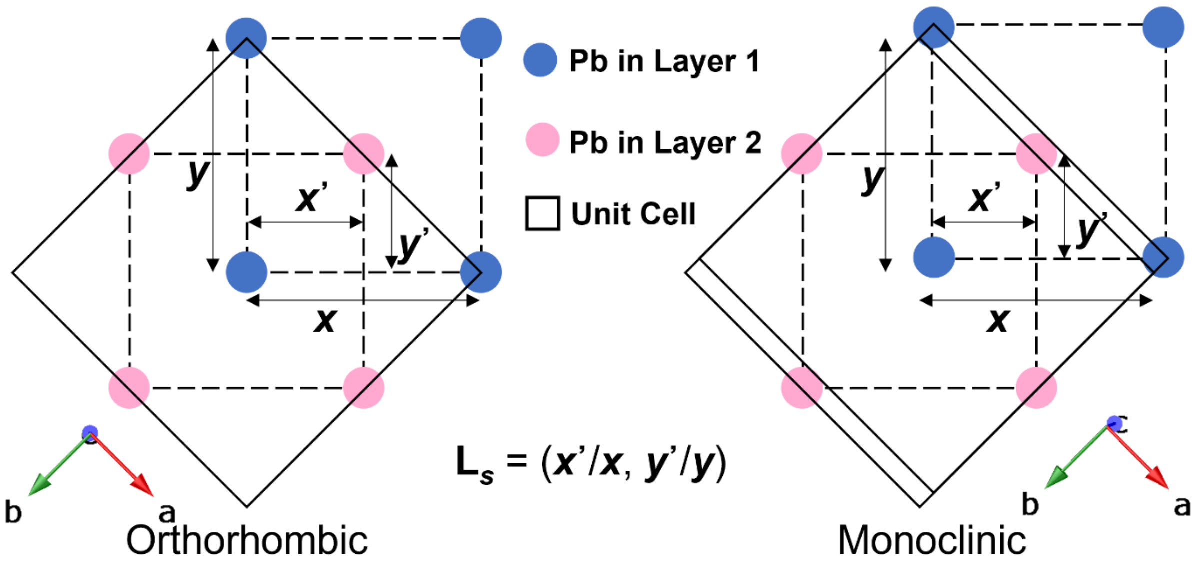

*Supplementary Figure 37: Diagram to illustrate the calculation of the **Ls.** Lead atoms in adjacent layers are represented by blue (layer 1) and pink (layer 2) spheres. The unit cell is outlined by the black solid square, and the in-plane Pb-Pb distance is indicated by black dashed lines.*

As shown in Supplementary Figure 37, **Ls** is the ratio of the in-plane displacements of the Pb atoms in Layer 2 (shown in pink) relative to those in Layer 1 (shown in blue), along the two in-plane directions, which have been denoted as *x* and *y*. Mathematically, **Ls** is expressed as (x'/x, y'/y), where x' and y' represent the projected in-plane distances of the atoms in Layer 2 relative to Layer 1 along the *x* and *y* directions.

Supplementary References

1. McNulty, J. A. & Lightfoot, P., Structural chemistry of layered lead halide perovskites containing single octahedral layers. *IUCrJ*, **8**, 485–513 (2021).

2. Smith, M. D., Jaffe, A., Dohner, E. R., Lindenberg, A. M. & Karunadasa, H. I., Structural origins of broadband emission from layered Pb-Br hybrid perovskites. *Chem. Sci.*, **8**, 4497–4504 (2017).

3. Deng,C., Zhou, G., Chen, D., Zhao, J., Wang, Y., Liu, Q., Broadband Photoluminescence in 2D Organic–Inorganic Hybrid Perovskites: $(C_7H_{18}N_2)PbBr_4$ and $(C_9H_{22}N_2)PbBr_4$. *J. Phys. Chem. Lett.*, **11**, 2934−2940 (2020)

4. Lemmerer, A. & Billing, D. G., Lead halide inorganic-organic hybrids incorporating diammonium cations. *CrystEngComm*, **14**, 1954–1966, (2012).

5. Lufaso, M. W. & Woodward, P. M., Jahn-Teller distortions, cation ordering and octahedral tilting in perovskites. *Acta Crystallogr., Sect. B:Struct. Sci.*, **60**, 10–20, (2004).

6. Robinson K., Gibbs G. V., Ribbe, P. H., Quadratic Elongation: A Quantitative Measure of Distortion in Coordination Polyhedra. *Science*, **172**, 567-570 (1971).

7. Guo, Y-Y., McNulty, J. A., Mica, N. A., Samuel, I. D. W., Slawin, A. M. Z., Bühl, M., Lightfoot, P., Structure-directing effects in (110)-layered hybrid perovskites containing two distinct organic moieties. *Chem. Commun.*, **55**, 9935–9938 (2019).

8. Guo, Y-Y., Yang, L-J, Biberger, S., McNulty, J. A., Li, T., Schötz, K., Panzer, F., Lightfoot, P.,. Structural Diversity in Layered Hybrid Perovskites, A_2PbBr_4 or AA′$PbBr_4$, Templated by Small Disc-Shaped Amines., *Inorg. Chem.*, **59**, 12858–12866 (2020).

9. Dohner, E. R., Hoke, E. T. & Karunadasa, H. I., Self-assembly of broadband white-light emitters. *J. Am. Chem. Soc.*, **136**, 1718–1721 (2014).

10. Li Y. Y., Lin C. K., Zheng, G. L., Cheng, Z. Y., You, H., Wang, W. D., Lin, J., Novel 〈110〉-oriented organic-inorganic perovskite compound stabilized by N-(3-aminopropyl)imidazole with improved optical properties., *Chem. Mater.*, **18**, 3463–3469 (2006).

11. Yang, L. Xuan, W., Webster, D., Krishnan Jagadamma, L., Li, T., Miller, D. N., Cordes, D. B., Slawin, A. M., Turnbull, G., Samuel, I. D. W., Chen, H. T., Lightfoot, P., Dyer, M. S., Payne, J. L., Manipulation of the Structure and Optoelectronic Properties through Bromine Inclusion in a Layered Lead Bromide Perovskite. Chem. Mater., 35, 3801–3814 (2023).

12. Ahmadian-Yazdi, M. R., Rahimzadeh, A., Chouqi, Z., Miao, Y. & Eslamian, M. Viscosity, surface tension, density and contact angle of selected PbI_2, $PbCl_2$ and methylammonium lead halide perovskite solutions used in perovskite solar cells. *AIP Adv.*, **8**, 025109, (2018).

13. Zhumekenov, A. A. et al. The Role of Surface Tension in the Crystallization of Metal Halide Perovskites. *ACS Energy Lett.,* **2**, 1782–1788 (2017).

14. Wang, S., Jiang, Y., Juarez-Perez, E. J., Ono, L. K. & Qi, Y., Accelerated degradation of methylammonium lead iodide perovskites induced by exposure to iodine vapour. *Nat Energy*, **2**, 1–8 (2017).

15. Grishko, A. Y., Eliseev, A. A., Goodilin, E. A. & Tarasov, A. B., Measure is Treasure: Proper Iodine Vapor Treatment as a New Method of Morphology Improvement of Lead-Halide Perovskite Films. *Chem. Mater.*, **32**, 9140–9146 (2020).

16. Solis-Ibarra, D., Smith, I. C. & Karunadasa, H. I., Post-synthetic halide conversion and selective halogen capture in hybrid perovskites. *Chem. Sci.*, **6**, 4054–4059 (2015).

17. Safdari,M., Svensson, P. H., Hoang, M. T., Oh, I., Kloo, L., Gardner, J. M., Layered 2D alkyldiammonium lead iodide perovskites: Synthesis, characterization, and use in solar cells., *J Mater. Chem. A*, **4**, 15638–15646 (2016).

18. Kim, S., Bae, S., Lee, S. W, Cho, K., Lee, K. D., Kim, H., Park, S., Kwon, G., Ahn, S-W., Lee, H-M., Kang, Y., Lee, H-S., Kim, D., Relationship between ion migration and interfacial degradation of $CH_3NH_3PbI_3$ perovskite solar cells under thermal conditions., *Sci. Rep.*, **7**, 1–9 (2017).

19. Chen X-G., Song, X-J., Zhang, Z-X., Zhang, H-Y., Pan, Q., Yao, J., You, Y-M., Xiong, R-G., Confinement-Driven Ferroelectricity in a Two-Dimensional Hybrid Lead Iodide Perovskite,. *J. Am. Chem. Soc.*, **142**, 10212–10218 (2020).

20. Li, T., Dunlap-Shohl, W. A., Reinheimer, E. W., Le Magueres, P. & Mitzi, D. B., Melting temperature suppression of layered hybrid lead halide perovskites via organic ammonium cation branching., *Chem. Sci.*, **10**, 1168–1175 (2019).
